# Confidence limits, error bars and method comparison in molecular modeling. Part 1: The calculation of confidence intervals

**DOI:** 10.1007/s10822-014-9753-z

**Published:** 2014-06-05

**Authors:** A. Nicholls

**Affiliations:** OpenEye Scientific Software, Inc., 9 Bisbee Court, Suite D, Santa Fe, NM 87508 USA

**Keywords:** Statistics, AUC, Virtual screening enrichment, Correlation coefficients, Linear regression, Error bars, Confidence intervals

## Abstract

Computational chemistry is a largely empirical field that makes predictions with substantial uncertainty. And yet the use of standard statistical methods to quantify this uncertainty is often absent from published reports. This article covers the basics of confidence interval estimation for molecular modeling using classical statistics. Alternate approaches such as non-parametric statistics and bootstrapping are discussed.

## Introduction: Error bars

When we report a number what do we mean by it? Clearly our intention is to convey information: after an experiment we think a property has a certain value; after this calculation our prediction of quantity X is Y. In reality, we know whatever number we report is only an estimate. For instance, we repeat an experiment to measure a partition coefficient between water and octanol five times and get an average, or we apply a computer model to a set of ten test systems and calculate a mean performance. In the former case, will a sixth measurement produce a similar number? In the latter case, do we know if the program will perform as well over a new test set? In other words, how do we know if these numbers are useful?

In statistics utility is about being able to say something concerning the *population* from a *sample*. Here *population* means “everything”, e.g. it could mean all members of a set, or all (infinite) repeats of an experiment. When we test predictive software we hope the average over a set of systems represents what we might get from the population of all possible test systems, including ones not yet imagined. For a physical property measurement we assume our experiments sample the possible range of small variations in conditions, what we call ‘random variables’, in an even and comprehensive way such that the ‘population’ of all such experiments is represented. In either case we know that we have only sampled, not enumerated all possibilities. As such there is an *uncertainty* in our number. In fact, without an assessment of this uncertainty, or a description of how to estimate it, what we have really delivered is a report, not a prediction; “we did X, followed by Y, and got Z”. In a completely general sense, i.e. from information theory, it can be shown that without at least some estimate of uncertainty a single value technically has no *information*—essentially because it is represented by a delta function in the probability distribution of possible values, which has a vanishing overlap with the actual distribution of values of the population. In reality a lone number *does* have some usefulness because we assign it a default sense of certainty from our experience. However such a sense can often be misleading, for instance our default may be wildly optimistic! A rigorous way of incorporating such prior knowledge is the Bayesian framework. Although Bayes approaches are very powerful and general, they lie outside the scope of this article. Interested readers should consider such excellent works as [1–5].

**Fig. 1:**
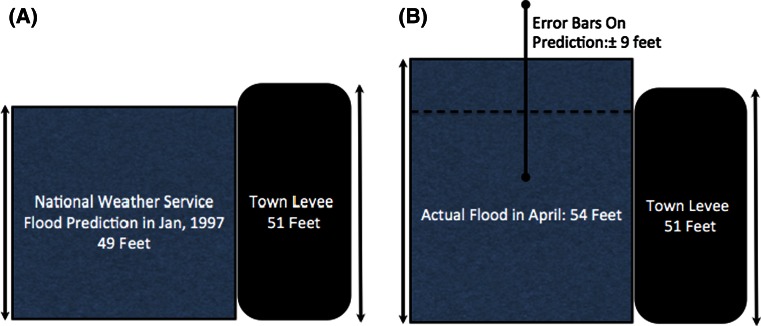
The predicted (**a**) and actual (**b**) flood levels at Grand Forks, North Dakota in 1997. The lack of *error bars* had catastrophic consequences

A classic case of the problems of reporting a single number without some sense of the range of possibilities is illustrated in Fig. 1. On the left was the prediction by the U.S. National Weather Service that the likely flood level of the Red River at Grand Forks, North Dakota in January of 1997 would be forty-nine feet. Based on this report the town levees were set at a protective fifty-one feet. What had not been included were the error bars on this prediction of plus or minus nine feet! The actually flood level in April of that year was fifty-four feet, and the cost of the ensuing devastation came to $3.5 billion. The error bars would have predicted the chance of a flood of this magnitude or worse at about one in three—substantial given the potential consequences. This *information* was not reported along with the prediction in part because of a fear that any apparent imprecision would lead to criticism of the forecast! Yet, to the people of Grand Forks the error bars were the key data.

Traditionally we express our uncertainty with an indication of our range of confidence. This can be by reporting a number with a plus-or-minus ‘error estimate’, or graphically by error bars attached to points or representative columns. We try to convey an expectation that the true value, the value of the ‘population’, lies within a given range: a probability assessment for the real value. Often this estimate is symmetric around our “best-guess”; we attempt to describe possible variation with a single, “plus-or-minus”, number. The reason this is common practice is that error in some variable *x* is often distributed according to a (symmetric) Gaussian function:

1 $$N\left( {\mu ,\sigma } \right) = (2\pi \sigma^{2} )^{ - 1/2} e^{{ - \frac{{(x - \mu )^{2} }}{{2\sigma^{2} }}}}$$

Here *μ* is the center of the function, our best guess at the average value, and σ is related to the width of the function, our uncertainty (a smaller σ means a narrower Gaussian, larger σ means a wider one). We only need to know σ to state what fraction falls within a given range of *μ*. The ubiquity of this description is a consequence of the famous “*Central Limit Theorem*” (*CLT*). The *CLT* says that if one samples from some distribution, no matter what *that* distribution looks like, the distribution of the *average* of that sample can be expected to look more and more like a Gaussian as the number of samples grows. This does not mean that the ‘true’ value is asymptotically approached; there might be an experimental bias away from the actual value. What the *CLT* tell us about is the reproducibility of the experimental setup we are using, i.e. it is concerned with precision, not accuracy.

The above description is typically taught in introductory classes to the scientific method, and experimentalists rarely forget it because reproducibility is their core concept. The same cannot be said for theoreticians. The presentation of error estimates, whether reasoned or derived, is rare in the field of computational chemistry. Perhaps this is because of the mistaken belief in the exactness of theory. Evidence for this would be a discernable inverse correlation between the level of theory in publications and the sophistication of any accompanying statistics. Or perhaps it is because practitioners see only a single number from a calculation that can be reproduced by rerunning the program. Of course, this belies the fact that small changes in either inputs to the program, programming presets or even the computer architecture or operating system can lead to great variability [6]. A third possibility is simply that knowledge is lacking. This author, for example, realized some years ago that he had only a very rudimentary knowledge of statistical assessment. It is to the latter possibility that this paper is addressed, to present, in the context of molecular modeling, how basic error bar evaluation and comparison should be done.

This goal turned out to be a considerable undertaking. The field of molecular modeling is diverse and complex, incorporating many levels of theory and physical approximation. Attempting to cover all eventualities is beyond the scope of a journal article. However, there are many common tasks and principles that would be of use if more widely known and this paper attempts to organize and present such a collection. In order to keep even that goal within reasonable bounds the statistics introduced here will be largely what is often referred to as “classical”. By classical we mean it is Frequentist and “parametric”. The term Frequentist refers to the school of statistics developed by Fisher, Pearson, Gossett, Neyman and others during the late 19^th^ century and first half of the 20^th^ century. It is based on the concept of reproducibility, of being able to imagine repeating events or experiments an arbitrary number of times. As such, quantities calculated from Frequentist approaches are “asymptotic”, by which is meant the key aspects are often just how many reproductions are necessary to give a certain level of confidence in a prediction. The Frequentist approach is often contrasted to the Bayesian approach, which is different in its focus on the past; what *did* we know and how does that help us make sense of what we did next. The advantage of the Bayesian approach is that it adapts more easily to the real world, e.g. some experiments we really cannot rerun. However, the Bayes formalism often requires numerical simulation and, in fact, really became popular only once computing power was widely available. The advantage of the Frequentist approach is a wealth of carefully constructed formulae that can be used to address all kinds of problems.

The availability of many of these formulae is due to the second part of the description of the work presented here, i.e. that the statistics are “parametric”. This term is often made synonymous with statistics that assume a Gaussian distribution of random variables, although more properly it applies to any approach where a functional form has been assumed for the distribution of some quantity, a functional form controlled by some “parameters”. In the case of Gaussians it is the center and the width, but there are other functional forms, e.g. Binomial, Poisson, Laplacian, Cauchy etc., with their own characteristics. Non-parametric, classical statistics do not make assumptions about the form of distributions and, as such, are more general. A few will be mentioned in this article. However, the focus will be on classical, Gaussian-based, statistics. The first reason is that classical statistics usually give a simple way to rapidly assess likely error and how this error decreases with sample size. More than other approaches, they can be “aids to thinking”, rather than magic boxes producing numbers. The second reason is to keep this report of manageable length.

Even with these decisions it has been necessary to split this paper into two parts, corresponding to the two definitive uses of confidence limits: comparison to fixed values, for instance the height of a levee, and comparison to other confidence limits: such as comparing prediction methods. Both uses are valuable; if your company gets a milestone payment if it identifies a one-nanomolar lead compound, then the accuracy of the measurement of that affinity is of some importance. If you are comparing two (or more) models of activity you will waste a lot of time and resources if you cannot tell which is more accurate. As such, the comparison of properties with associated confidence intervals will be described in a subsequent paper, with the focus here on the estimation of a single error bar.

Given these restrictions the structure of the paper is as follows:

1. Basic principles
  - Standard terms
  - The Gaussian distribution
  - One- or two-tailed significance
  - Long tails
  - The test statistic, *t*
  - The origin of the square-root in asymptotic error
  - Reporting data, box plots
  - The error in the error
2. Small sample size effects
  - The Student *t* distribution
  - *p* values and the Student test statistic
3. Useful analytic forms for error bars in modeling
  - Probabilities
  - Area under the (ROC) curve (AUC)
  - Virtual screening enrichment
  - Linear regression (straight-line fit) properties
  - Pearson’s correlation coefficient, *r*
4. Asymmetric error bars
  - Probabilities
  - Area under the (ROC) curve (AUC)
  - Virtual screening enrichment
  - RMSE
5. Combining errors from different sources
  - General formulae and examples
  - The general error formula
  - Estimating unknown contributions to error
  - Assessing adding noisy systems to test sets
  - Variance-weighted averages with examples
  - Weighted averages of variances
6. Bootstrapping error bars
  - Introduction
  - Limitations
  - Advantages

## Basic principles

### Standard terms

Error bars are a graphical depiction of a confidence interval; a range within which we expect some value to fall with a certain probability given what we currently know. Suppose we make *N* observations, *x*_*i*_, and calculate the average:

2 $$\overline{x} = \frac{1}{N}\mathop \sum \limits_{i = 1}^{N} x_{i}$$

The *standard deviation* (SD) is defined as the square root of the average squared difference from the mean. It is often represented by the Greek symbol (lower case) sigma, σ. However this is strictly meant for the SD of the *population*, as described above. The SD of the *sample* is represented by the letter “*s*”, and defined as:

3 $$s_{N} = \sqrt {\frac{1}{N - 1}\mathop \sum \limits_{i = 1}^{N} (x_{i} - \overline{x} )^{2} }$$

Note that the averaging of the sum of squares uses (*N* − 1) not *N*, the number of observations. This is necessary because the formula uses the *sample* mean, $\overline{x}$, not the *population* mean (“*μ*”). This is generally explained as due to the (*N* − 1) *degrees of freedom* in the expression for *s*_*N*_. The concept of degrees of freedom occurs a lot in classical statistics, and is typically represented by the symbol ν. It basically means, “How many independent samples from a distribution *really* occurred”. In the above example we can see that we could derive any one measurement from the mean and the rest of the values, e.g.

4 $$x_{N} = N\overline{x} - \mathop \sum \limits_{i = 1}^{N - 1} x_{i}$$

As such, there are really only (*N* − 1) variables in the equation for *s*_*N*_. This explanation always seemed mysterious to this author! As such, “Appendix 1” includes a simple proof that using (*N* − 1) gives an estimate of the SD that is unbiased, i.e. in the limit of large sample sizes the sample mean will approach the population mean by being slightly larger or slightly smaller with equal likelihood. In many cases it is not obvious how many degrees of freedom there are; sometimes approximations are employed that give fractional degrees!

Another widely used term is the ‘*variance*’. This term can be flexible; typically it refers to the square of the standard deviation, although sometimes it can also refer to the standard deviation divided by the number of samples. In the former case it refers to the intrinsic property of how widely spread are the measurements. In the latter case it refers to the spread of likely values of the *average* of those measurements. In this case it is the square of the *standard error* (SE) of the mean. The standard deviation and standard error are related by:

5 $$SE = SD/\sqrt N$$

Given $\overline{x}$ and *s*_*N*_, the usual prescription for 95 % confidence limits to a quantity, *x*, is:

6 $$E\left[ x \right] = \overline{x} \pm \frac{{1.96s_{N} }}{\sqrt N } = \overline{x} \pm 1.96SE$$

Here “*E*” stands for “*Estimate*”. There is a lot in this simple formula: where did the square root of N come from? Why “1.96” and why 95 %? The 95 % is completely arbitrary and should be treated with suspicion! It comes from R. A. Fisher, one of the founders of Frequentist statistics. Fisher decided that if nineteen times out of twenty the difference in yield between two differently treated fields of cabbages was less than would be expected due to random variation, then there was no real difference in the two treatments. This sense of ‘real’ as being defined by being more unusual than “one in twenty” now pervades statistics, so much so that there are real concerns as to the problems it causes [7–9]. For instance, if the consequences of being outside the predicted range are small then one in twenty may be a perfectly acceptable risk. If billions of dollars and lives are at stake, as in the Red River example in Fig. 1, then perhaps it is not. There is also a problem when this concept is invoked relative to deviation from a “null” model, i.e. perhaps a simpler model. In this case the interpretation in terms of the probability one method is better than another can be subtle [9]. Finally, if multiple comparisons are made, i.e. if one is actively searching amongst many methods, then some will appear significantly different by random chance. Despite these issues, “*p* values” of 0.05 are almost inescapable.

### The Gaussian distribution

**Fig. 2:**
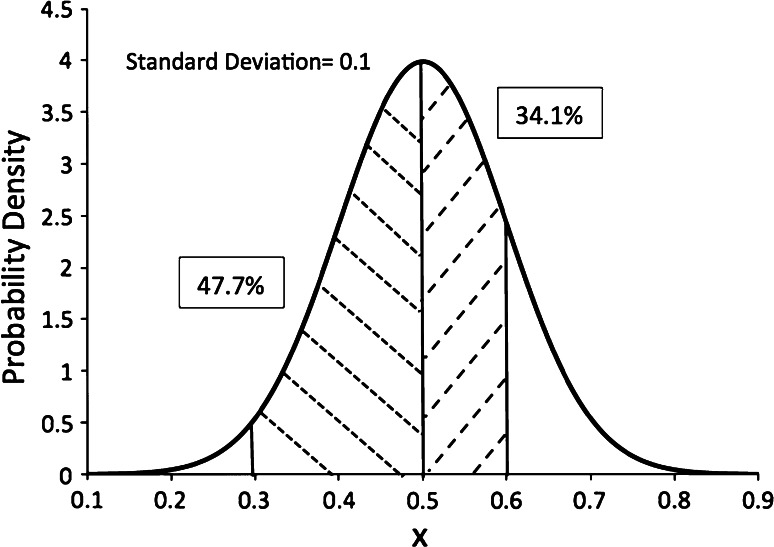
A Gaussian centered at 0.5 with standard deviation 0.1. The one-sided percentage under one and two standard deviations from the mean (*center*) is indicated. The y-axis is not probability, but probability density

Suppose we have a property with a standard deviation, σ, of 0.1 units and an average of 0.5 units for a set of measurements of a property *x*. Our Gaussian distribution of what we know about *x* appears in Fig. 2.

The *y*-axis in Fig. 2 is the ‘probability density’ not probability. Probability density tells us the ‘amount’ of probability in the local vicinity of a value of x, i.e.

7 $$p\left( {x \in \left[ {x - 0.5\delta ,x + 0.5\delta } \right]} \right) = pdf\left( x \right) * \delta$$

The function *pdf*(*x*) has to be positive but does not have to be less than one, which at first can be confusing. Within one standard deviation of the center lies 68.2 % of the probability density and 95.4 % within two standard deviations. It can be shown that 95 % of the area lies between ±1.96σ, which is close enough to two standard deviations that the two are often used interchangeably, i.e. two standard deviations is used to represent 95 % of the likelihood.

### One- or two-tailed significance

An important distinction needs to be made here as to the “sided”-ness of areas under a Gaussian. The 95 % confidence limits that are usually mentioned refer to the possibility of a value being larger or smaller than a given range. But suppose we are interested in whether a value is larger than a given value? For that we do not have to consider the lower range—our interest is “one-tailed”, i.e. only concerns one tail of the distribution function. For instance, in the above example, there is only a 2.5 % chance the actual value is greater than 0.7. One-sided comparisons go with questions such as, “Is quantity A greater than a value X”, or “Is quantity A less than a value Y”, but not both (in which case the two-tailed distribution is required). A focus of classical statistics, possibly to its detriment, is whether two things are different. In such tests we are agnostic as to which might be better and which worse, only if they are distinguishable. As such, the 95 % range and its association with roughly two standard deviations from the mean is appropriate. However, when we are asking a more specific question: is drug *A* *worse* than drug *B*, is treatment *C* *better* than no treatment, we are asking a one-tailed question. As this issue is more germane to the comparison of quantities, i.e. to confidence limits on differences of properties, further discussion will be postponed until the second paper of this series.

### Long tails

Much of the criticism of classical statistics concerns the tails of the Gaussian distribution not being accurate. For example, Taleb and others [10, 11] have pointed out that the distribution of returns on stock investment is Gaussian (i.e. random) for short time intervals but that rare events (“Black Swans”) appear much more frequently than expected. Taleb co-founded an investment vehicle, “*Empirica Capital*”, based on this principle, i.e. designed to lose money when the stock market was behaving in a regular, “Gaussian” manner, and yet to win big when it deviated from this behavior. There is considerable work in the area of unlikely events and their distributions, so-called “extreme-value” distributions such as the Gumbel, Fréchet or Weibull distributions [12]. In addition, we will consider the most applied “long-tailed” function, the Student *t*-function, shortly.

### Test statistic, *t*

The number of standard deviations used to test for a probability of something happening is usually referred to as *t*, the ‘*test statistic*’. It is called that because it is what we use to test a difference between an observed value and the expected (mean), scaled by the standard error, i.e. we expect 95 % of the time:

8 $$\frac{{\sqrt N \left\lceil {x - \overline{x} } \right\rceil }}{\sigma } < t_{95\,\% }$$

This equation is very basic as it relates to the probability of seeing a (two-tailed!) difference of a given size. Note it is the size of the effect scaled by the standard error, not the standard deviation. The origin of the square root of *N* in the standard error is considered next.

### The origin of the square root in asymptotic error

The fact that the error in an average goes down with the square root of the number of observations was not always appreciated. Examples of it *not* being known can be dated back to the *Trial of the Pyx*, 1282 AD [13]. The *Trial* was a test designed by the English Royal Mint to check for unlawful deviations in the weight of the King’s coinage and it was assumed such variation would be linear with the number of coins tested. Thus an unnecessarily large tolerance was assumed allowing the unscrupulous but mathematically astute to ‘game’ the King’s system, at some peril naturally! Even today it is at the root of many misunderstandings of published data [14].

So why does a square root appear in the error of the average? All that is required is to know that the probabilities for independent events multiply. Suppose we want to estimate the variation of the average of *N* quantities, but let us assume the average is known to be zero, i.e. *μ* = 0. It makes no fundamental difference but the derivation is simpler. Then the variance is simply:

9 $$Var(\left\langle x \right\rangle ) = \left\langle {\left( {\mathop \sum \limits_{i = 1}^{N} x_{i} /N} \right)^{2} } \right\rangle = \left\langle {\mathop \sum \limits_{i = 1}^{N} x_{i}^{2} /N^{2} } \right\rangle + \left\langle {\mathop \sum \limits_{i = 1}^{N} \mathop \sum \limits_{j \ne i}^{N} (x_{i} x_{j} )/N^{2} } \right\rangle$$

Now, the first term is just:

10 $$\left\langle {\mathop \sum \limits_{i = 1}^{N} x_{i}^{2} /N^{2} } \right\rangle = \frac{1}{N}\left\langle {\mathop \sum \limits_{i = 1}^{N} x_{i}^{2} /N} \right\rangle = \frac{{\sigma^{2} }}{N}$$

The second term must be equal to zero, because the different measurements of *x*_i_ are independent, i.e.

11 $$\left\langle {x_{i} x_{j} } \right\rangle = 0$$

One way to look at this is that the N^2^ terms for the variance of the average of N things reduces to just N terms because of measurement independence, and so instead of a dependence on √(N^2^), we get √N.

### Reporting data, box plots

Although 95 % is a standard for a confidence interval, there are variations worth knowing. The first is that “1.96” is often simply replaced with “2.0”, a ‘two-sigma’ error bar, since this makes very little difference (95.4 % compared to 95.0 %). However, this leads to error bars that are based on the number of sigmas, not a percentage. So, for instance, a one-sigma error bar (not uncommon) contains 68.2 %, roughly two-thirds, of expected outcomes; a three-sigma error bar (unusual) contains about 99.7 %. It is important a researcher is clear as to which is being presented—especially if the smaller one-sigma error bars are reported. No report is complete unless the meaning of presented error bars is explicitly recorded.

**Fig. 3:**
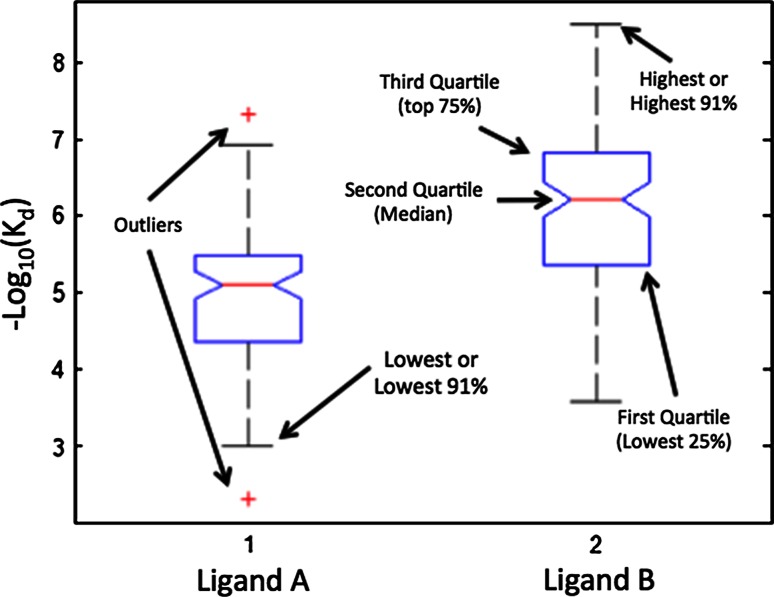
A typical “*Box*” plot containing much more information than the standard *error-bar* plots

Secondly, we have described above the difference between the intrinsic variance, or *standard deviation*, which is a measure of the spread of measurements, and the extrinsic variance, or *standard error*, which is a measure of the accuracy of the mean. The common confusion between these led Tukey to introduce the “box plot”. An example is shown in Fig. 3. Tukey’s plots were ‘*non*-*parametric*’, i.e. were not intended to rely on the data following a Gaussian. As such his ‘boxes’ were meant to represent ‘ranges’ of data, e.g. the top box is the range from the median (Q2) to the third quartile (Q3), i.e. the 25 % of measurements greater than the median, the bottom box the lower 25 % (Q1). In Tukey’s original designation the “whiskers” represent the nearest data points to 1.5 * (Q3 − Q1) of the median in either direction. However, since its introduction box plots have evolved in several ways; only the meaning of the size of the boxes is standard. The whiskers can represent the maximum and minimum observations, or a given % (typically 91 % and 9 % of all measurements). In the latter case, measurements that lie outside this range are represented by crosses, as in the outliers depicted for the affinity of ligand A in Fig. 3. More importantly, for our purposes, were the introduction of ‘notches’ around the median. These can be used to represent the standard error, i.e. the extrinsic property of the collection of measurements. Notches, then, are equivalent to the more standard error bars, where as the boxes describe the variation within the set of measurements as well as the extrema. As such, box plots are a rich description of the measurements of properties.

Why are box plots non-parametric, i.e. why the ‘median’ and “quartiles”, rather than the mean or SD? The answer lies in Tukey’s interest in “Robust Statistics” [15]; a field he helped to create. Robust statistics attempts to address the problems that outliers can cause to traditional, parametric, Gaussian-based statistics. For instance, a single outlier can shift the mean (or SD) of a set of measurements an arbitrary amount; the mean (or SD) is ‘fragile’ with respect to a single measurement. Contrast this to the median (or quartile) where adding a single value, no matter how extreme, can move the median (quartile) only to an adjacent measurement. Ripley nicely describes Tukey’s investigations and those that followed in robust statistics [16].

### The error in the error

As the variance plays such a key role in classical statistics an obvious question might be as to how we calculate *its* variance. At least as far as Gaussian statistics goes, this is an easy question to answer:

12 $$\sigma^{2} = \overline{\sigma }^{2} \pm \sqrt 2 t_{95\,\% } \frac{{\bar{\sigma }^{2} }}{{\sqrt {N - 1} }}$$

A derivation of this result can be found in “Appendix 2”. Notice that the error bounds are given for the variance, not the standard deviation. Because we have to take a further square root to get the error limits for the standard deviation they will not be symmetric! We will consider asymmetric error bars in some detail in a later section, but as an illustration consider the Gaussian depicted in Fig. 2 with a SD of 0.1. Let us assume that this is an estimate derived from fifty observations, then:

13 $$\begin{aligned} &\sigma^{2} = (0.1)^{2} \pm \sqrt 2 t_{95\,\% } \frac{{(0.1)^{2} }}{{\sqrt {50 - 1} }} \hfill \\ &\sigma^{2} \in \left[ {(0.1)^{2} - \sqrt 2 * 2\frac{{\left( {0.1} \right)^{2} }}{{\sqrt {50 - 1} }},\left( {0.1} \right)^{2} + \sqrt 2 * 2\frac{{(0.1)^{2} }}{{\sqrt {50 - 1} }}} \right] \hfill \\ &\sigma^{2} \in \left[ {0.6 * (0.1)^{2} ,1.40 * \left( {0.1} \right)^{2} } \right] \hfill \\& \sigma \in \left[ {0.077,0.118} \right] \hfill \\ \end{aligned}$$

Where we have set the *t*_95 %_ to 2.0. As predicted, the 95 % confidence limits are asymmetric, the lower limit differing from the “best guess” by more than the upper limit.

The variation of the standard deviation or, more properly, of the variance is of use. Consider that common measure, the root-mean-square-error or *RMSE*. The *RMSE* is a standard deviation; it is the σ of some property prediction because it describes the expected variation of that property. We talk about the *RMSE* of affinity prediction or solvation or solubility estimation, for example. Equation 12 tells us how to estimate the error in our assessment. For instance, if a paper quotes an *RMSE* for affinity prediction of 2.0 *kcal/mol* for a test system with fifty data points:

14 $$\begin{aligned} &RMSE^{2} = \overline{RMSE}^{2} \pm \sqrt 2 t_{95\,\% } \frac{{\overline{RMSE}^{2} }}{{\sqrt {N - 1} }} \hfill \\ &RMSE^{2} = 2.0^{2} \pm \sqrt 2 t_{95\,\% } \frac{{2.0^{2} }}{{\sqrt {50 - 1} }} \hfill \\ &RMSE^{2} \in \left[ {4 - \frac{8\sqrt 2 }{7},\,4 + \frac{8\sqrt 2 }{7}} \right] \hfill \\ &RMSE \in \left[ {1.54,\,2.37} \right] \hfill \\ \end{aligned}$$

Here we set *t*_95 %_ to 2.0 again. Now, suppose we had only eight data points and we repeated the calculation. The range for the RMSE squared would come to:

15 $$\begin{aligned} &RMSE^{2} \in \left[ {4 - \frac{8\sqrt 2 }{\sqrt 7 },\,4 + \frac{8\sqrt 2 }{\sqrt 7 }} \right] \hfill \\ &RMSE^{2} \in \left[ { - 1.33,\,8.28} \right] \hfill \\ \end{aligned}$$

But the lower limit of a squared quantity cannot be negative! So what has gone wrong? The problem is that the distribution of expected variation of an averaged quantity is Gaussian, by the *CLT*, when the number of observations is *large*. It is an asymptotic observation, not a universal one. The properties of the variation for *small* samples can be quite different. We shall return to a more complete description of the error bounds on *RMSE* in the section on asymmetric confidence intervals, but first consider what “large” means for sampling from Gaussian distributions.

## Small samples

### The Student *t* distribution

William Gossett worked at Guinness as one of the first industrial statisticians. He was aware of the standard Gaussian statistics that Karl Pearson (of the Pearson *r* coefficient), Fisher and others were developing. However, his work revolved around small numbers of samples, not the large data sets that Pearson had in his biometric work or Fisher at his agricultural station. Gossett was confronted with problems such as the quantity of barley of a particular strain to use in fermentation or a particular size of fermentation vat, or at what temperature to run the process. Here the number of such experiments he could affect was in the single digits, not hundreds or thousands. He applied to be an intern with Pearson for a year and wrote two papers (published under the name Student to abide by Guinness’ internal policies) that changed the world of statistics by rigorously showing what the expected distribution of averages should look like for small samples [17]. That function is known as the Student *t*-distribution:

16 $$f\left( t \right) = \frac{{\Gamma \left( {\frac{{\upnu + 1}}{2}} \right)}}{{\sqrt {\upnu \uppi } \varGamma \left( {\frac{\nu }{2}} \right)}}\left( {1 + \frac{{t^{2} }}{\upnu}} \right)^{{ - \frac{{\upnu + 1}}{2}}}$$

**Fig. 4:**
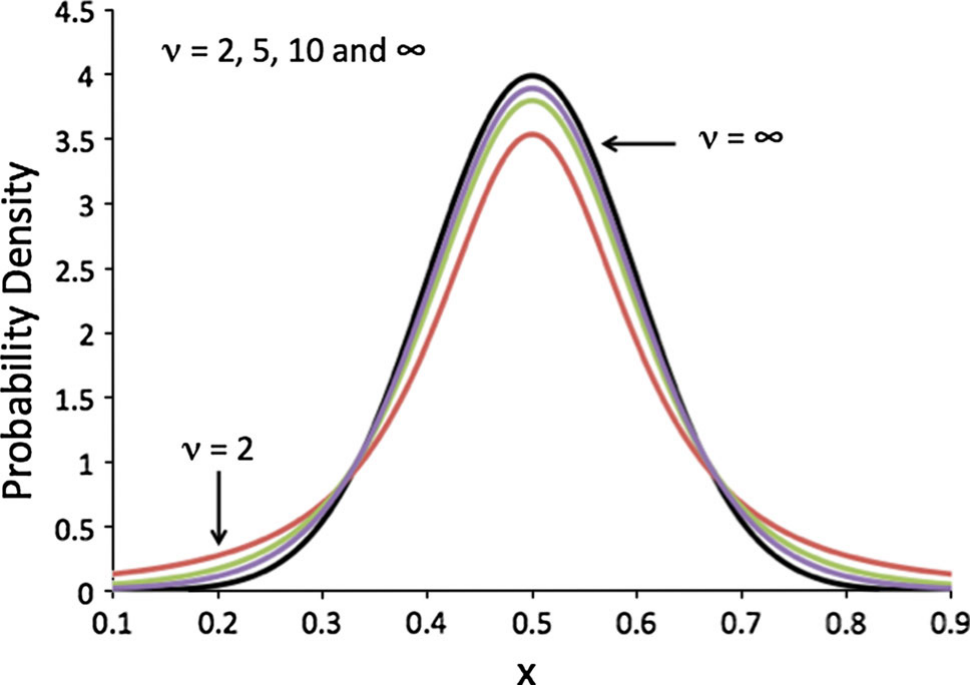
The Student *t*-distribution for different degrees of freedom, *ν* (number of samples = *ν* + 1). When *ν* is large the function approaches a Gaussian but has longer ‘tails’ for small values of *ν*

Here the symbol *Γ* represents the gamma function (equivalent to the factorial function for integer values). Figure 4 illustrates it for different ν values.

### *p* values and the Student test statistic

As the number of degrees of freedom, *ν*, increases the function looks more and more like a Gaussian, but for small *ν* it has wider tails. This means the 95 % confidence band is significantly larger. As such, the factor “*1.96*” for 95 % of the area under the Student-*t* function needs to be replaced with a bigger number—significantly bigger for very small *ν*. Table 1 shows some example values.

**Table 1:** Table showing the 95 % Student *t* test statistic for different numbers of data points, *N*

N (*ν* + 1)	*t*_95 %_
2	12.71
3	4.30
4	3.18
5	2.78
10	2.26
20	2.09
50	2.01
100	1.98
∞	1.96

As can be seen, you need about twenty samples before the inaccuracy in the prefactor of 1.96 is less than 10 %. Consider the case where a standard deviation is being estimated from a measurement done in triplicate—the *t* statistic is more than twice what one would expect for large sample sizes!

The ramifications of Student’s work were slow to materialize but were eventually recognized as fundamental to practical statistics in industrial settings. It also illustrates one of the issues with “classical” statistics, i.e. its reliance on look-up tables. That is inevitable because the functions that describe the different probability distributions are not common outside of statistics. These days, however, it is easy to find such tables on-line.

One further aspect of the Student-*t* that has only become appreciated in recent years is that it can also be used to improve the robustness of linear regression [18]. This is because the long tails of the Student function better tolerates outliers, i.e. the “unlikelihood” of an outlier does not outweigh the “likelihood” of “in-liers” as much.

## Useful analytic forms for error bars in modeling

We present here some known and some new results for error bars of typical measures of importance in computational chemistry, namely, (1) the probability of an event, (2) the area under the curve (AUC) for receiver operator characteristics (ROC), (3) virtual screening enrichment, (4) Pearson’s *R* ^2^ and (v) linear regression. Analytic results may seem old-fashioned when modern computing power can simulate distributions, e.g. via bootstrapping, but they can be invaluable when the primary data is not available. In addition, they allow us to think about the contributions to the error terms in a way that simulations do not. Finally, as will be discussed below, there are occasions when the prior knowledge they represent can be helpful in producing more robust estimates.

### Probabilities

The mathematics described above for the estimation of a confidence limit is very basic and it is not always obvious how it is to be applied. Take the simple example of a probability *p*, arrived at from observing an outcome *X* a total of *m* times out of *N*, e.g. a docking program places molecules within 2 Å of the crystal structure *m* times out of *N*. What is the error bar on *p*? Suppose we think of the observation *X* as having a value *γ*, where *γ* is equal to 1.0 when we see the event and 0.0 when we do not. Then *p* is the average value of “*γ*”. If we looked to calculate the variance of *γ* we would obtain:

17 $$\begin{aligned} var\left( \gamma \right) = & \frac{1}{N - 1}\mathop \sum \limits_{i = 1}^{N} \left( {\gamma_{i} - \bar{\gamma }} \right)^{2} = \frac{1}{N - 1}\mathop \sum \limits_{i = 1}^{N} \left( {\gamma_{i} - p} \right)^{2} \\ = & \frac{1}{N - 1}\left[ {\mathop \sum \limits_{i = 1}^{N} \gamma_{i}^{2} + \mathop \sum \limits_{i = 1}^{N} p^{2} - 2\mathop \sum \limits_{i = 1}^{N} \gamma_{i} p} \right] = \frac{1}{N - 1}\left[ {Np + Np^{2} - 2Np^{2} } \right] \\ = & \frac{N}{N - 1}\left( {p - p^{2} } \right) \approx p - p^{2} \\ \end{aligned}$$

I.e. we have a very simple formula for the variance of the probability, and hence for the SD and 95 % confidence limits:

18 $$\sigma \approx \sqrt {p - p^{2} }$$

19 $$p = \frac{m}{N} \pm t_{95\,\% } \sigma /\sqrt N$$

We can translate this into error bounds on an integer (count) by multiplying Eq. 19 though by *N*.

20 $$n = Np = m \pm t_{95\,\% } \sigma \sqrt N$$

Typical political polls have a sample size of N = 1,000. If there are two candidates, *p* is likely to be about 0.5, for which σ is then roughly also 0.5 from Eq. 18. The fraction error is then about 1/√*N* ≈ 0.03, which is the origin of the oft-quoted three percent margin of error. If *p* ^2^ is much smaller than *p* then the error bars on *p* are roughly ±2√(*p*/*N*). Translated to error bounds on the number of observations, *m*:

21 $$n = Np = m_{observed} \pm t_{95\,\% } \sqrt {Np(1 - p)} \approx m_{observed} \pm 2\sqrt {m_{observed} }$$

I.e. a quick estimation of the expected 95 % range of a number of observations is twice the square root of the number of observations. This formula is only appropriate when p is small, yet it should be noted that this is also the range in which one has to worry about error bars not straying into nonsensical regions, i.e. an error bound on a probability should not suggest a value that is less than zero. This condition is examined later in this article. One final observation on Eq. 21 is that it is mysteriously free of the sample size, *N*! As such, as long as an event is rare, i.e. *p* is small, knowledge of the total number of events is not required.

### Area under the (ROC) curve (AUC)

**Fig. 5:**
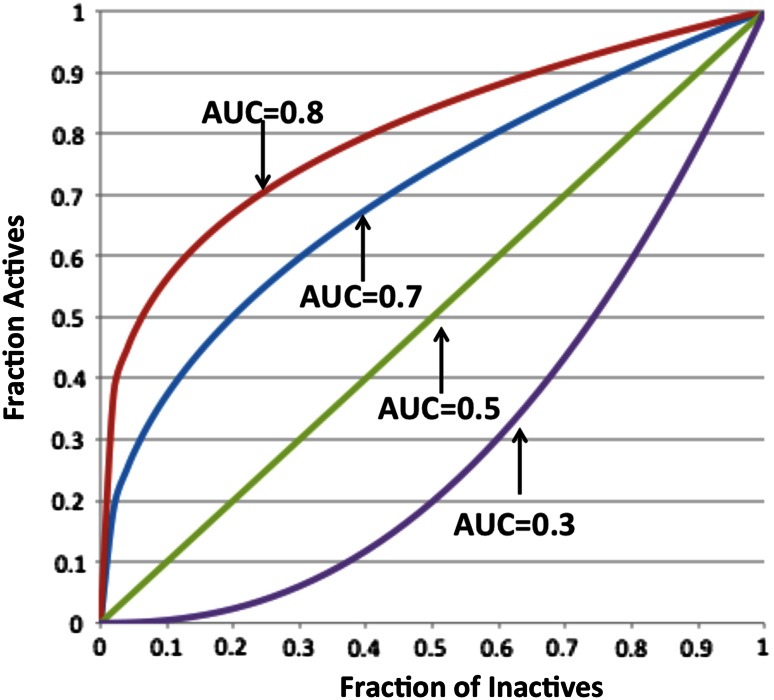
Illustration of receiver operator characteristic curves, with annotations of their area under the curve

A popular metric of virtual screening is the AUC, or area under the curve, where the curve is the ROC or receiver operator characteristic curve. Despite its odd name, which came from its origins in radar detection, it is simply a plot of *Y*, the fraction of true results (e.g. active compounds) observed to have a property (e.g. docking score) greater than some threshold, *T*, as a function, *X*, of the fraction of false results (e.g. inactives) which also are lower than this threshold. As *T* is varied from the highest value any molecule possesses to the lowest, a curve is swept out from the origin (0,0) to the point (1,1). If all the actives are seen before any inactives then the area under this curve (essentially two sides of the unit square) is 1.0. When the actives are randomly distributed with respect to the inactives the AUC will, on average, be 0.5 as the ROC ‘curve’ will be a line from (0,0) to (1,1). Figure 5 illustrates the concept.

The ROC AUC is equivalent to the probability a randomly chosen true event is ranked higher than a randomly chosen false one, i.e. the higher the AUC the greater the ability of the property to distinguish true from false. In what follows ‘true’ will mean active, e.g. an active molecule, a correctly docked molecule etc., whereas ‘false’ will mean an inactive molecule, an incorrectly docked molecule etc. A subtlety arises as to how ties are managed. The simplest prescription, followed here, is to count a tie as one half of its normal contribution.

The expected accuracy of the AUC will depend on the number of actives and the number of inactives. Consider each active in turn. It contributes to the AUC by the fraction of inactives for which it ranks higher. Since this contribution is a probability, accuracy of this property will depend on the number of inactives. We then combine the probability of this active with the similar probability for all other actives. This average of probabilities will have its own distribution, the tightness of which will depend on the square root of the number of actives. Thus there are two sources of error. In a later section we shall more generally consider the situation of multiple contributions to error but this is an example of just such, i.e. error from the finite number of actives and from the finite number of inactives.

If the variance for each active was strictly proportional to the probability, *p*, we could merely average over all actives to obtain the net variance. However, since the variance depends on *p* ^2^, not just *p*, we need to know the *distribution* of *p* across all actives to calculate its average, i.e. we cannot calculate the expected error bars without knowing the shape of the ROC curve. If we have the primary data we know this and can use the formula for the total error in the AUC first proposed by Delong et al. [19]:

22 $$Var_{Total} = \frac{{Var(p_{active} )}}{{N_{active} }} + \frac{{Var(p_{inactive} )}}{{N_{inactive} }}$$

Here *N*_{*active*/*inactive*}_ is the total number of {actives/inactives} and the variance, *Var*(*p*_{*active*/*inactive*}_) is the variance associated with the probability each {active/inactive} scores higher than a randomly chosen {inactive/active} averaged over all {actives/inactives}, i.e.

23a $$Var(p_{active} ) = \frac{1}{{N_{active} - 1}}\mathop \sum \limits_{i = 1}^{{N_{active} }} \left( {p_{i, active} - AUC} \right)^{2}$$

23b $$Var(p_{inactive} ) = \frac{1}{{N_{inactive} - 1}}\mathop \sum \limits_{j = 1}^{{N_{inactive} }} \left( {p_{j, inactive} - (1 - AUC)} \right)^{2}$$

Note the (1 − *AUC*) factor in Eq. 23b. If the AUC is the probability an active scores higher than an inactive then the reverse property, i.e. the probability an inactive scores higher than an active, must simply be (1 − *AUC*). Equation 22 is an example of combining different contributions to produce a net error.

But what of the case when the primary data is not available, i.e. we do not know the shape of the ROC curve? There are two approaches in this situation. The first is to average over *all* possible ROC curves that have that AUC. This sounds like a formidable challenge but Cortez et al. [20] did just that to arrive at a complicated result using combinatorics. The second approach is to assume what the ROC curve looks like, i.e. to use a ‘typical’ curve with the same AUC, e.g. as introduced by Hanley et al. [21]. By assuming a simple form for the scores of actives and inactives (an exponential) they derived a analytic form that resembles many ROC curves:

24 $$Y = X^{{\frac{1 - AUC}{AUC}}}$$

The curves in Fig. 5 were produced from this equation. The expected standard error of an AUC of this form is:

25a $$\begin{aligned} &w = AUC_{observed} \hfill \\ &Var(active) = \frac{{w^{2} (1 - w)/(1 + w)}}{{N_{active} }} \hfill \\ \end{aligned}$$

25b $$Var(inactive) = \frac{{w(1 - w)^{2} /(2 - w)}}{{N_{inactive} }}$$

25c $$AUC = w \pm t_{95\,\% } \sqrt {\frac{{w^{2} (1 - w)/(1 + w)}}{{N_{active} }} + \frac{{w(1 - w)^{2} /(2 - w)}}{{N_{inactive} }}}$$

Note that Eq. 25b can be obtained from Eq. 25a simply by replacing *w* with (1 − *w*), by analogy with swapping *AUC* for (1 − *AUC*) in the Delong formula. These equations are derived from integrating across the distribution form proposed by Hanley. See [22] for a complete derivation.

**Fig. 6:**
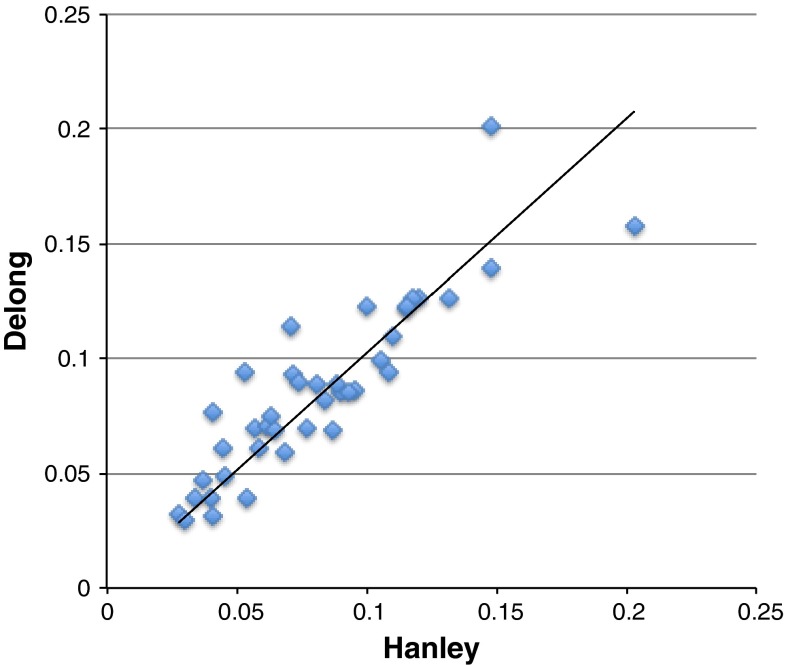
The expected standard error in the AUC for forty systems from the DUD dataset using the docking program FRED v1.2, as calculated by the Hanley and Delong formulae. The drawn line is X = Y. The Hanley result used only the AUC value while Delong used the actual form of the ROC curve, i.e. the primary data

ROC curves never look perfectly like those in Fig. 5. Figure 6 shows a comparison of the Hanley equation to that from Delong on a set of results using a docking program over the DUD dataset [23]. Even though typical ROC curves from this study are very ‘non-ideal’, the correspondence is strikingly good. In fact, the two points that disagree most are for systems for which there are fewest actives (only eight and sixteen), i.e. where the expected error in error from the Delong estimation should be large and where the result from an analytic formula might be better behaved than the direct result, i.e. from the primary data. This possibility is considered in more depth when we examine the concept of bootstrapped estimates.

It is worth noting that this is not the first time the accuracy of the Hanley approach has been examined. Cortez et al. [24] compare their exhaustive enumeration of ROC curves with the Hanley result and that from Delong’s formula and found any improvement was marginal.

But what about the *t*-*statistic*, the “1.96” value we use to determine the 95 % confidence intervals? From Gossett we know that for small samples we have to use his tables. Do we use *N*_*active*_, the number of actives, or *N*_*inactive*_, the number of inactives? When there are multiple contributions to an error a widely used approach is the Welch–Satterthwaite formula [25, 26]:

26 $$\upnu_{effective} \approx \frac{{\left( {\mathop \sum \nolimits_{i = 1}^{m} Var_{i} /N_{i} } \right)^{2} }}{{\mathop \sum \nolimits_{i = 1}^{m} \frac{{\left( {Var_{i} /N_{i} } \right)^{2} }}{{\upnu_{i} }}}}$$

Here there are *m * different sources of error adding to the total variance, *Var*_*i*_ is the variance (standard deviation squared) of each contribution to the error and *N*_*i*_ the number of data points associated with that error and ν_i_ the number degree of freedom, typically (*N*_*i*_ − 1). We know all these quantities from either the Delong and Hanley formulae. Using the latter we can eventually arrive at:

27 $$\upsilon_{effective}^{AUC} = \frac{{\left( {\alpha N_{inactive} + \beta N_{active} } \right)^{2} }}{{(\alpha N_{inactive} )^{2} /(N_{active} - 1) + (\beta N_{active} ) ^{2} /(N_{inactive} - 1)}}$$

where,

$$\alpha = \frac{AUC}{1 + AUC};\quad \beta = \frac{1 - AUC}{2 - AUC}$$

Given that the variances from the actives and decoys are roughly equal, if the number of decoys is much larger than the number of actives Welch–Satterthwaite would give *ν*_effective_ close to (*N*_*active*_ − 1). If the number of actives is roughly equal to the number of inactives the number of degrees of freedom will be closer to the sum of both.

### Virtual screening enrichment

A common complaint against the use of the AUC curve is that it does not measure the quantity of interest, i.e. the segregation of actives to the very top of the list, the ‘early’ enrichment. Such claims are misinformed, as the AUC is a reliable estimate of early performance; in fact, averaged over many systems it is a better estimate of early enrichment than artificial measures that have been ‘designed’ to reflect this quantity [27]. This is because the AUC uses all the data and so it is more statistically reliable (√N is larger). For instance, it can be shown that the AUC is more robust to the inclusion of “false false positives”, i.e. compounds that are assumed inactive but are actually active [27]. The second reason is that although a single AUC value *may* mislead as to early performance, e.g. the ROC curve might have a sigmoidal shape where the early enrichment is poor but some less relevant middle enrichment makes the AUC look good, *averaged* sets of ROC curves tend to look very ‘canonical’, i.e. have Hanley shapes [27]. Such averages of AUC correlate very well with measures of early enrichment, but with much better statistical properties.

Despite the above observation, the field is attracted to measures of early enrichment, typically defined as the ratio of the percent actives recovered when a given percent of the database has been screened to the expected percent of actives if they were indistinguishable from inactives. For instance, if 10 % of the database has been screened and 20 % of all actives have been found then the enrichment is 2.0. This deceptively simple formula has a flaw that makes it undesirable as a metric—it depends on the ratio of inactives to actives [28]. It makes little sense to choose a metric that depends on an arbitrary, extrinsic aspect of the system, e.g. the relative numbers of active and decoys. Metrics should be *intrinsic*, e.g. how well does this docking program work, not how well does this docking program work given this ratio of actives to inactives—something that will clearly not be known in advance in a prospective application.

To illustrate this, suppose we have ten actives and 990 inactives and we look at the enrichment at 10 %. We would expect (at random) one active in that top 10 %, i.e. top 100, by chance. If we see all ten actives we would claim an expected maximum enrichment of ten, i.e. the reciprocal of 10 %. If we have 90 inactives, we would similarly expect one active in the top 10 %, i.e. ten compounds, and again if all the top ten were actives we would achieve the maximal expected enrichment. If we have forty inactives, however, while we would expect only one active at random in the top five, i.e. top 10 %, even if all the top five are actives the enrichment is now only five, not ten. In general, if *R* is the ratio of inactives to actives.

28 $$Maximum\;Enrichment = \hbox{min} \left( {\frac{1}{Enrichment\;Fraction}, 1 + R} \right)$$

Note this saturation effect has nothing to do with the total number of actives and inactives, just their ratio and it clearly gets worse at smaller enrichment percentages. At 1 % enrichment you need R > 99, for 0.1 %, R > 999 and so on. And, of course, this saturation effect is noticed before the enrichment limit is reached. One approach would be to simply make sure inactives are always in great excess. Better, though, is to redefine the enrichment as the fraction of actives found when a given fraction of *inactives* have been found. This metric, which we will call the *ROC* enrichment [28], is essentially the ratio of *Y* to *X* of a point on the AUC curve. It is independent of *R* and is an *intrinsic* property of the method. It also allows for straightforward calculation of the expected error because both the fraction of actives and the fraction of inactives can be looked upon as probabilities, for which we can calculate variances.

Suppose we set:

- *A* = total number of actives
- *I* = total number of inactives
- *f* = fraction of inactives observed at a threshold T
- *g* = fraction of actives observed at the same threshold
- *e* = ROC enrichment
- *S* = *dg*/*df* = slope of the ROC curve at point (*f*, *g*)

By our definitions,

29 $$e(f) = g/f$$

Now, the finite number of actives and inactives means that there can be error in *e*(*f*) due to variance in *g*, the fraction of actives, and also from the ‘error’ in *f*, the fraction of inactives. To see this, imagine we keep the actives constant but introduce a different set of inactives. Then the fraction *g* of actives at fraction *f* of inactives will likely change, i.e. the expected error in the AUC needs to include terms that involve both the number of actives, *A*, and the number of inactives, *I*.

Next, rather than considering the variance of *e*, the enrichment, consider the ratio of *e* to the maximum possible enrichment, i.e. *1/f*. This number, *ef*, must run from zero (no actives found) to one (all actives found) and hence is like a probability. In fact, it *is* a probability, the probability an active is found before a fraction *f* of inactives is found. As such, we expect the variances of the contributions from *f* and *g* will look like the variances for probabilities, i.e.

30a $$var(g) = \frac{{g\left( {1 - g} \right)}}{A}$$

30b $$var(f) = \frac{{f\left( {1 - f} \right)}}{I}$$

To see how these ought to be combined we need to know how the variance of a function of a variable depends on the variance of that underlying variable—i.e. how *f* depends on *g*. The standard result, which we illustrate in “Appendix 3”, is that for a function *H* of random variable *x*, we have:

31 $$var\left( {H(x)} \right) \approx \sigma_{x}^{2} \left( {\frac{\partial H}{\partial x}} \right)^{2}$$

i.e. the variance of the function of *x* is scaled by the square of the rate of change of the function with respect to *x*. For our case, the rate of change of *g*, the fraction of actives, with *f*, the fraction of inactives, is simply the slope, *S*, of the ROC curve at *f*. So we have:

32 $$\begin{aligned} &var(ef) = var(g) + S^{2} var(f) \hfill \\ &var(ef) = \frac{{g\left( {1 - g} \right)}}{A} + S^{2} \frac{{f\left( {1 - f} \right)}}{I} \hfill \\ \end{aligned}$$

We can approximate *S* from the values of *g* at (*f* ± δ). Alternatively, we can look to the Hanley formula for a typical ROC curve. If we do this we arrive at a simple formula for *S*:

33 $$S = \frac{g}{f}\left( {1 + \frac{\log (e)}{\log (f)}} \right)$$

As such, the total variance of enrichment is:

34a $$var(e) = \frac{1}{{f^{2} }}\left( {\frac{{g\left( {1 - g} \right)}}{A} + \left( {\frac{g}{f}\left( {1 + \frac{\log (e)}{\log (f)}} \right)} \right)^{2} \frac{{f\left( {1 - f} \right)}}{I}} \right)$$

and the expected 95 % error bars on an enrichment value are:

34b $$enrichment(f) = e \pm t_{95\% } \frac{1}{f}\sqrt {\frac{{g\left( {1 - g} \right)}}{A} + \left( {\frac{g}{f}\left( {1 + \frac{\log (e)}{\log (f)}} \right)} \right)^{2} \frac{{f\left( {1 - f} \right)}}{I}}$$

However, this formula uses the variables of the *ROC Enrichment*. Most published enrichments are in the less preferable form of “enrichment as a fraction of the database”. It is possible to translate from one form to the other. The details are presented in “Appendix 4”. If *R* is the ratio of inactives to actives:

35a $$var\left( E \right) = var\left( e \right)\frac{{\left( {1 + R - E} \right)^{4} }}{{\left( {1 + R} \right)^{2} R^{2} }}$$

35b $$E = E \pm t_{95\;\% } \frac{{\left( {1 + R - E} \right)^{2} }}{{\left( {1 + R} \right)R}}\sqrt {\text{var} (e)}$$

It is shown in “Appendix 4” how to derive the variance of the ROC Enrichment purely in terms of the quantities of traditional enrichment. As with AUC, the degrees of freedom necessary to calculate the *t*-statistics can be derived from the Welch–Satterthwaite formula (Eq. 26), with the variances of the active and inactive fractions in the place of the variances for the actives and inactives. As with the AUC it is likely that inactives are in excess and so ν_effective_ is approximately (N_active_ − 1).

### Linear regression (straight-line fit) properties

Although there are obvious drawbacks in assuming a linear relationship between predictor and predicted, it can also make a lot of sense. It is often the simplest model beyond the average of a set of experimental values (a “null” model that itself ought to be applied more often). In addition, although we deal with complex systems, we often assume that while one variable cannot explain an effect entirely, everything left out in the explanation might be proportional to what is left in, i.e. that our key variable merely needs to be scaled. Examples of this are simplified molecular polarization models wherein the induced field is assumed linearly proportional to the field from the static molecular charges, i.e. the assumption is made that subsequent induction caused by this ‘first order’ induction can be captured by a scaling factor. Methods such as Generalized Born [29] use this *ansatz*. The scaling of charges to mimic polarization in force fields is a similar example (it is presumed polarization energies are proportional to the increase in Coulombic interaction between molecules with scaled dipole moments). The approach is widely used in other sciences; for example in simulating the properties of stellar bodies it is sometimes easier to model the electrodynamics than the magnetohydrodynamics [30]. Similar *ansatz* occur in nuclear physics (e.g. the Bethe–Weizaecker formula of the liquid drop model), quantum mechanics (e.g. functional construction in Density Functional Theory), statistical mechanics (e.g. liquid theory virial expansion cutoffs) and solid-state physics (e.g. effective interaction potentials of quasi-particles).

Even though models are not necessarily linear, it is typical that linear regression quality is often used as a measure of model quality. Given the widespread use of linear regression, it is surprising that straightforward estimates of the likely errors in the slope and intercept are seldom published.

Suppose:

$$y = \alpha + \beta x$$

Here the variable *y* represents our estimation of some property using *x*. The estimate of *β* that minimizes the mean-square error is:

36 $$\beta = \frac{{\sigma_{xy} }}{{\sigma_{x} }}$$

Where,

37 $$\sigma_{xy} = \frac{1}{N - 1}\mathop \sum \limits_{i = 1}^{N} (x_{i} - \bar{x})(y_{i} - \bar{y})$$

The term *σ*_*xy*_ is called the ‘covariance’ since it measures the degree to which *x* and *y* vary similarly, “co-vary”, from their respective means. The covariance is intimately related to the concept of correlation, e.g. Pearson’s correlation coefficient, which will be considered in the next section. It can be shown that if the error in the estimation of *y* by *x* is distributed as a Gaussian and is independent of x, then the variation of the slope will also be a Gaussian, and that its variance will be:

38 $$var(\beta ) = \frac{{\mathop \sum \nolimits_{i = 1}^{N} \left( {y_{i} - \hat{y}_{i} } \right)^{2} /(N - 2)}}{{\mathop \sum \nolimits_{i = 1}^{N} \left( {x_{i} - \bar{x}} \right)^{2} }}$$

The accent on *y* means it is the linear prediction of *y* for a given *x*. As such the numerator in this equation is just the mean square error of the linear fit, but where we are dividing by *N* − 2 instead of *N*. The reason for this, as might be anticipated, is that there are two degrees of freedom in the prediction, i.e. the slope and the intercept. The 95 % confidence interval for the slope, *β*, is then:

39 $$\beta = \hat{\beta } \pm t_{95\,\% } \sqrt {var(\beta )}$$

Here the *t*-statistic uses *N* − 2 as the degrees of freedom. The variance of the intercept is simply a scaled version of the variance of the slope:

40 $$var(\alpha ) = var(\beta )\mathop \sum \limits_{i = 1}^{N} x_{i}^{2} /N$$

And:

41 $$\alpha = \hat{\alpha } \pm t_{95\,\% } \sqrt {var(\alpha )}$$

Of course, the same “small numbers” conditions apply to averages from Gaussian samples (such as *α* and *β*), i.e. more extreme variation should be expected as per the Student *t*-distribution if *N* is small.

An obvious question to ask is whether the variability of slope and intercept are independent of each other, because if the slope changes then we would expect the offset to alter, and if the offset changes the slope will have to adjust so that the best fit line goes through the middle of the data. This has some very real consequences for applying a linear regression model. For instance, if both the slope and intercept are used in a formula we cannot assume the combined error comes from independent sources. Consider predictions made with the linear model. When a point is further away from the center of the data it will be more sensitive to the slope and when near the center more sensitive to the offset. There is a relatively simple formula that accounts for both, i.e. accounts for their covariance, to give 95 % confidence interval as a function of *x*, the predictor value. The classical formula that includes the variance of both and their covariance is:

42 $$y(x) = \hat{y} \pm t_{95\,\% } \sqrt {(x - \bar{x})^{2} Var(\beta ) + \frac{1}{N}\mathop \sum \limits_{i = 1}^{N} \left( {y_{i} - \hat{y}_{i} } \right)^{2} /(N - 2)}$$

An approximation to this formula that brings out its essential features is:

43 $$y(x) = \hat{y} \pm t_{95\% } \frac{RMSE}{\sqrt N }\sqrt {1 + \frac{{12(x - \bar{x})^{2} }}{{L^{2} }}}$$

Here, the *RMSE* is the root mean square error of the linear fit over all *N* points, and L is the *range* of *x*, i.e. the maximum *x* minus the minimum *x*.

There are three items to notice in this formula:

(i) At the center of the data range the expected error in *y* is the average error divided by *√N*, as if all *N* points were variations the mean *x* value.
(ii) Away from the center the error is magnified by a hyperbolic term
(iii) This magnification is scaled by the inverse of the range of the data.

**Fig. 7:**
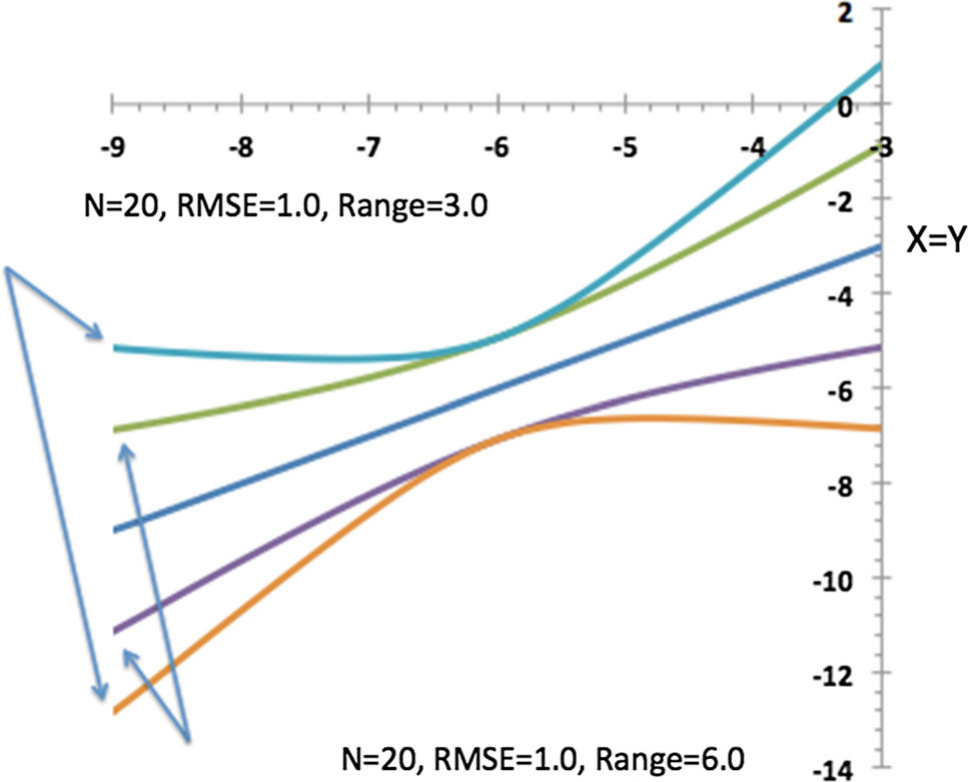
A graph showing the expected 95 % confidence limits for predictions from a linear fit. The *central line* is “X = Y” and represents the achieved fit to twenty data points (*N* = 20). The *two lines* bracketing “X = Y” show the 95 % confidence limits for predictions made from this *line* when the net RMSE = 1.0 across a data range of 6.0 U, about a median of x = −6.0. The *two outer lines* represent the same limits but for a data range of 3.0, about a median of *x* = −6.0 again

Figure 7 illustrates these points, in particular the dramatic effect of the range on the expected error.

A real-world example of this occurs in the estimation of vacuum-water transfer energies. These estimations are very useful in testing theories of solvation but the direct measurement of these energies is difficult. Indirectly one can use the combination of vapor pressure and solubility, whether from the liquid or solid form, i.e. the transference from vacuum to water can be thought of as a two stage process: (1) from vapor to solid or liquid form (minus the vapor pressure), then (2) from solid or liquid form to solvated form (solubility).

Vapor pressure depends on temperature via the Clausius–Clapeyron equation:

44 $$\ln \left( P \right) = - \frac{{\varDelta H_{vap} }}{RT} + C$$

Typically this equation is used to extrapolate for vapor pressure *P* to a temperature of interest. As such, errors in the slope and the intercept can both play a role in the estimation of the vapor pressure that goes into estimation of the solvation energy [31]. de Levie [32] has a similar example for the estimation of room temperature rate constants, along with more in-depth analysis of this common problem.

### Pearson’s correlation coefficient, *r*

Perhaps the most common metric seen in papers is the correlation coefficient between two variables. Introduced by Pearson [33], *r*, or more usually *r* ^*2*^, is a measure of how closely two variables follow a linear relationship. The formula for *r* is very simple:

45 $$r = \sigma_{xy} /(\sigma_{x} \sigma_{y} )$$

Where σ_xy_ is the covariance as defined above (Eq. 37). If *x* and *y* vary independently then *σ*_*xy*_ is equal to zero. If *x*, or *x* plus an offset, is directly proportional to *y* then *r* is equal to ±1, depending on the sign of proportionality.

The square of *r* can be interpreted as the “fraction of variance explained by the predictor variable”. i.e.

46 $$r^{2} = \frac{{\mathop \sum \nolimits_{i} \left( {\hat{y}_{i} - \bar{y}} \right)^{2} }}{{\mathop \sum \nolimits_{i} \left( {y_{i} - \bar{y}} \right)^{2} }}$$

In this equation the numerator is the variance of the linear fit, while the denominator is just the variance of the original *y*.

With *r* in hand, calculating the slope and intercept of the best-fit line is simply:

47a $$\beta = r\frac{{\sigma_{y} }}{{\sigma_{x} }};$$

47b $$\alpha = \bar{y} - \beta \bar{x}$$

Another nice result is:

48 $$\frac{{(y - \bar{y})}}{{\sigma_{y} }} = r\frac{{(x - \bar{x})}}{{\sigma_{x} }}$$

I.e., if we center both *x* and *y* by their mean values and then ‘normalize’ each by their standard deviations, there is no offset just a slope between them that is just *r*!

**Fig. 8:**
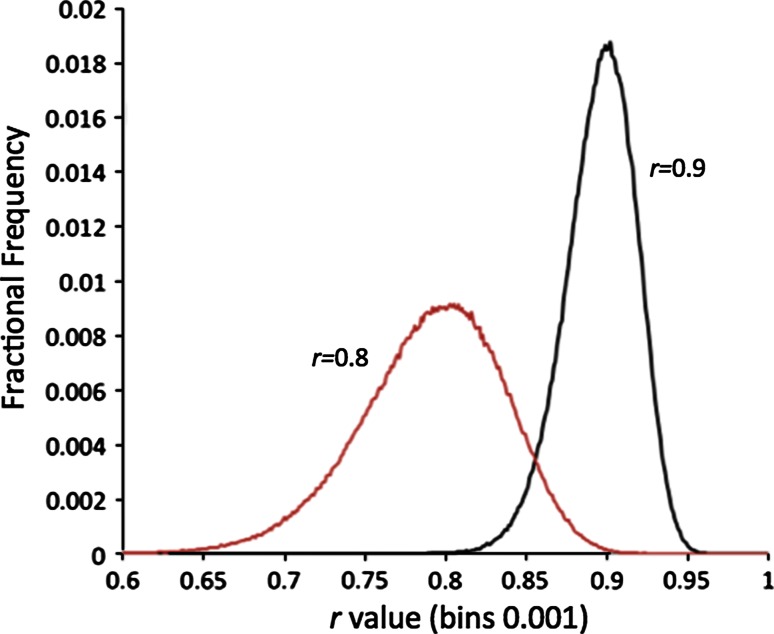
Distributions of *r*-values with most likely values of 0.8 and 0.9, produced by random sampling of the correlation coefficient of 50 points pairs of *x* and *y,* evenly spaced between 0.0 and 4.0

However, when we try to estimate *var*(*r*) we face a problem. Pearson’s *r* has a range (−1, +1), not (−∞, +∞). So how can we talk about a Gaussian distribution for *r* if this extends to plus and minus infinity? In fact, this is a common problem for any measures that are limited, including ones considered above. Figure 8 shows what a distribution looks like of correlation coefficients of 0.8 and 0.9, produced by adding noise into a correspondence. As can be seen, the distributions are asymmetric, particularly for 0.9 as the upper bound of 1.0 exerts its effect.

One solution to this problem is to transform variables. As we saw above, there is a simple, if approximate, method to calculate the variances of functions of random variables. If a transformation is not bounded, the *CLT* guarantees that the distribution of the average of that transformed variable will (in the limit of many samples) be a Gaussian. Consider the function:

49 $$F\left( r \right) = \frac{1}{2}ln\frac{1 + r}{1 - r}$$

This is known as the Fisher transform and it transforms the range [−1, 1] to [−∞, +∞]. Fisher used it to show that the transformed correlation coefficient, *F,* has a Gaussian distribution with a standard deviation close to one, making its confidence limits simple to calculate [34].

The procedure to calculate confidence intervals for *r* is then to take the values that represent the confidence limits for *F* and back-transform them to obtain limits for *r*. Fisher also showed that the standard error decreases with respect to √(N − 3), rather than √N. As such, the prescription for error bars for *r* is:

1. Calculate *r*
2. Calculate the value of *F*(*r*)
3. Add and subtract {*t*-statistic*/√*(*N*−3)} to obtain confident limits for *F.*
4. Back-transform these confidence limits into *r*-values. These are the *r*-value confidence limits, and will be asymmetric.

The back-transform function is:

50 $$r(F) = \frac{{e^{2F} - 1}}{{e^{2F} + 1}}$$

As an example, suppose we have calculated *r* = 0.9 (*r* ^2^ = 0.81) for ten points.

$$r = 0.9 \to F(r) = 0.5\ln \left( {1 + 0.9/1 - 0.9} \right) = 1.472$$

$$\text{var} \left( {F(0.9)} \right) = \frac{1}{N - 3} = \frac{1}{7} = 0.143$$

$$F = 1.472 \pm \sqrt {0.143} t_{95\,\% } = [0.617, 2.327]$$

$$r(F) = \left[ {\frac{{e^{2 * 0.617} - 1}}{{e^{2 * 0.617} + 1}}, \frac{{e^{2 * 2.327} - 1}}{{e^{2 * 2.327} + 1}}} \right]$$

$$r(F) = [0.55,0.98]$$

$$r^{2} = [0.30, 0.96]$$

This range is probably much larger than many people would expect for what seems, on the face of it, to be a pretty good correlation!

Another simple statistical feature of *r* is its significance level, i.e. what is the probability that, for this number of points, we should see an r equal to this value or greater by random chance? If we follow the prescription above we could find the confidence interval for *r* = *0.0* and see if our *r*-value lies within it:

51 $$r \in \left[ {\frac{{e^{{\frac{{2t_{95\,\% } }}{{\sqrt {N - 3} }}}} - 1}}{{e^{{\frac{{2t_{95\,\% } }}{{\sqrt {N - 3} }}}} + 1}},\frac{{e^{{ - \frac{{2t_{95\,\% } }}{{\sqrt {N - 3} }}}} - 1}}{{e^{{ - \frac{{2t_{95\,\% } }}{{\sqrt {N - 3} }}}} + 1}}} \right]$$

Note that the range for *r* = 0.0 is symmetric. If *N* is large this approximates to:

52 $$r \in \left[ { - t_{95\,\% } /\sqrt {N - 3} ,t_{95\,\% } /\sqrt {N - 3} } \right]$$

i.e.

53 $$\left| {r_{significant} } \right| = t_{95\,\% } /\sqrt {N - 3} ,$$

A more accurate formula can be arrived at by considering the exact distribution for *r* = 0. This leads to:

54 $$r_{significant} = \frac{{t_{95\,\% } }}{{\sqrt {N - 2 + t_{95\,\% }^{2} } }}$$

As *N* gets larger so does this threshold *r*, meaning we can be more confident a result is not random if we have more points. For the example above with so few points we have a larger *t*_95 %_ of 2.262 leading to

$$r_{significant} = \frac{2.262}{{\sqrt {8 + 2.262^{2} } }} = 0.62$$

This, again, may be a surprise, i.e. if there are only ten data points even an *r* of, say 0.6 (<0.62) is not statistically significant at the 95 % level.

Finally, researchers should be aware of the difference between the *sample* and the *population* versions of *r*. As we saw earlier, there can be a bias in a statistical quantity, such as the standard deviation, for finite sampling size. This happens to be true for Pearson’s *r*. If we let the population (e.g. infinite sampling size) *r* be *ρ* then an unbiased estimator (bias of order 1/*N* − 1) for large *N* is:

55 $$\langle \rho \rangle = r\left( {1 - \frac{{1 - r^{2} }}{{2\left( {N - 1} \right)}}} \right)$$

This equation tells us the expected value of the *population r* is smaller (absolute sense) than the *sample* correlation coefficient *r*. In the above example, where *r* = 0.9 and *N* = 10, this formula suggests a small correction of *r* to 0.89. The correction is larger if *r* or *N* are smaller.

Pearson’s *r* is central to many disciplines; it has become the *sine qua non* of “discovery”, i.e. is there an effect or not. As such it is important to understand its limitations, for instance the expected error, the sensitivity to outliers, and what it tells us about the underlying causes of the correlation. This topic will be explored further in the follow-on article in which we consider the comparison of *r*-values.

It should be noted that there are at least two other popular measures of correlation, namely the Kendall’s tau and Spearman’s rho. Tau measures the preponderance of correct orderings within a list, e.g. what proportion of list pairs do we see ranked in the correct order, whereas the Spearman’s rho is a rank order facsimile of Pearson’s *r*, i.e. values X and Y are replaced with ranks of each variable, i.e.

56 $$\rho_{xy} = \frac{{\mathop \sum \nolimits_{i = 1}^{N} (rnk.x_{i} - av \cdot rnk)(rnk \cdot y_{i} - av \cdot rnk)}}{{\sqrt {\mathop \sum \nolimits_{i = 1}^{N} (rnk \cdot x_{i} - av \cdot rnk)(rnk \cdot x_{i} - av \cdot rnk)} \sqrt {\mathop \sum \nolimits_{i = 1}^{N} (rnk \cdot y_{i} - av \cdot rnk)(rnk \cdot y_{i} - av \cdot rnk)} }}$$

If the distributions of errors are roughly Gaussian, and the relationship is linear, then there are formulae that can interconvert between *r* and τ and ρ and provide error estimates for each [35]. Notably, simply dividing the (*N* − 3) term in equations dealing with the confidence intervals for *r* by the constant 1.06 gives equivalent significance values for ρ. Also, τ and ρ are much more robust (outliers can rearrange at most 1/*N* of the total number of rank comparisons) and they can find any monotonic relationship, not just linear correspondences.

There also exist a wide range of “pseudo” *r*-squared quantities that can be used for categorical variables, such as McFadden’s, Efron’s, Cox and Snell’s, and the Nagelkerke or Cragg and Uhler’s [36]. These feature as analogs of Pearson’s *r* but for logistic regression, i.e. when what we want to predict is essentially binary, e.g. active or inactive. The process of logistic regression is appropriate to many aspects of computational chemistry; however, there are few applicable insights into its error analysis from classical statistics and so it falls outside the scope of this article.

Another class of *r*-values attempts to account for the number of parameters in the model, for instance the “adjusted” *r*-squared of Theil [37]:

57 $$\overline{{R^{2} }} = 1 - (1 - R^{2} )\frac{N - 1}{N - 1 - \# parameters}$$

Similarly, there is a variant of McFadden’s pseudo *r*-squared that penalizes parameters. Such variants are purposed towards model comparison and not estimating quality of correlation. Furthermore, there are reasons to prefer other tests for comparing parameterized models, such as Fisher’s *F*-test [38], or tests that include parameter penalties from information theory, e.g. Akaike’s Information Content (AIC) or Schwarz’s Bayes Information Criteria (BIC) [39, 40].

## Asymmetric error bars

As we saw for Pearson’s *r*, error bars can be asymmetric when there are fundamental bounds to the confidence limits. The way forward in such cases is to transform to a variable that is unlimited, and hopefully with an error distribution that is more symmetric and Gaussian. We then calculate error bars for this new variable, and finish by transforming these error limits back to the original variable. In this section this process is examined for the quantities of interest considered above.

### Probabilities

Probabilities can only range from zero to one. The transformation typically applied is the well-known *logit* function:

58 $$f(p) = log\frac{p}{1 - p}$$

As required, the range of this function is (−∞, +∞) for an input that is (0,1). To use this to calculate effective error bars we need two addition formulae, the derivative of this function with respect to *x* and its inverse.

59a $$\frac{df}{dp} = \frac{1}{p(1 - p)}$$

59b $$f^{ - 1} (p) = \frac{1}{{1 + { \exp }( - f)}}$$

Thus the prescription is as follows:

1. Calculate the standard error of the input p, *SE*(*p*), i.e. *sqrt*(*p*(1 − *p*)/*N*)
2. Calculate *f*(*p*)
3. Multiply *SE*(*p*) by (*df*/*dp)* to get the standard error *SE*(*f*).
4. Calculate *f* ± *t*_95 %_ * *SE*(*f*).
5. Back-transform these two values to obtain the confidence interval in *p*

This process can be put into a single, succinct formula:

60 $$p_{95\,\% } = \left[ {\frac{p}{{p + \lambda \left( {1 - p} \right)}},\frac{p}{{p + \lambda^{ - 1} (1 - p)}}} \right]$$

Where,

61 $$\lambda = e^{{t_{95\,\% } /\sqrt {p\left( {1 - p} \right)N} }}$$

In the limit of large N this reproduces the expected “Gaussian” limits.

### The Area Under the (ROC) Curve (AUC)

As stated previously, an AUC for an ROC curve can be interpreted as the probability a randomly chosen active has a higher score than a randomly chosen inactive. As such, we simply follow the same multi-step procedure as above for probabilities, but where we substitute the formula for the standard error of the AUC, i.e. Eq. 25c, rather than the standard error for the probability. As such, there isn’t such a nice analytic formula as for probability but the procedure is straightforward, i.e. we have to follow the prescription of transformation, calculate the transformed limits, and then back transform to obtain the limits in terms of a probability.

As an example, suppose we have an AUC of 0.9 with many inactives yet only ten active compounds. First we transform the AUC with the *logit* equation:

62 $$f = \log \left( {\frac{p}{1 - p}} \right) = \log \left( {\frac{0.9}{0.1}} \right) = 2.20$$

Equation 25c gives the standard error:

63 $$SE_{AUC} \left( {0.9} \right) \approx \sqrt {\frac{{0.9^{2} \left( {1 - 0.9} \right)/\left( {1 + 0.9} \right)}}{{N_{active} }}} = 0.065$$

If we were to build a confidence limit of two standard deviations we would obtain: [0.9 – 2 * 0.065, 0.9 + 2 * 0.065], i.e. [0.77,1.03]. Thus the upper limit would exceed one! So instead, we multiply the standard error by the formula in Eq. 59a, i.e.

64 $$SE_{f} (2.20) = 0.065 * \frac{1}{{p\left( {1 - p} \right)}} = 0.065 * \frac{1}{{0.9\left( {1 - 0.9} \right)}} = 0.72$$

The limits, assuming a *t*-statistic of 2.0, are [2.20 – 2 * 0.72, 2.20 + 2 * 0.72], i.e. [0.76, 3.64]. Transforming these values back with Eq. 59b, we arrive at:

65 $$AUC \in \left[ {\frac{1}{{1 + \exp \left( { - 0.76} \right)}},\frac{1}{{1 + \exp \left( { - 3.64} \right)}}} \right] = [0.68, 0.97]$$

We note that the lower limit of 0.68 is substantially lower than the untransformed limits of [0.77, 1.03], but conversely the upper limit of 0.97 is lower and now sensible. The actual width of the confidence interval is almost the same, i.e. 0.26 untransformed and 0.29 transformed; it has just swung from higher to lower. We see similar behavior in Fig. 8 for the distribution of Pearson’s *r*-value of 0.9, where the upper 95 % confidence bound is only half the width of the lower 95 % confidence bound.

### Virtual Screening Enrichment

As described above, one can define an enrichment quantity that is bounded by zero and one, i.e. the ROC Enrichment scaled by the percent of inactives. This can also be treated as a probability; it is the probability that an active is seen before a given percent of inactives. As such, this quantity can be treated by the standard procedure, i.e. transform the scaled measure, scale the variance using the derivative of the *logit* function, calculate the confidence limits in the transformed space, back transform and finally scale to a ROC Enrichment by dividing by the percent of inactives. The question of the number of effective degrees of freedom follows a similar treatment as with AUC, i.e. the Welch–Satterthwaite formula, Eq. 26, whose elements are the individual variances of actives and inactives and their respective counts. If the number of decoys is much larger than the number of actives then the latter is used as the effective number of degrees of freedom.

If the more traditional definition of enrichment is being used then we should first transform to the equivalent ROC enrichment numbers. This is important because we cannot extract a simple probability from the traditional enrichment value because of saturation. Saturation means that the apparent probability we see, i.e. the probability an active is found in a given percent of the database, is dependent on other factors, such as the ratio of actives to inactives. Transforming to the ROC enrichment gives a pure probability for which confidence limits can be established. These can then be transformed to traditional enrichment numbers. The formulae for these transformations can be found in “Appendix 4”.

### RMSE

**Fig. 9:**
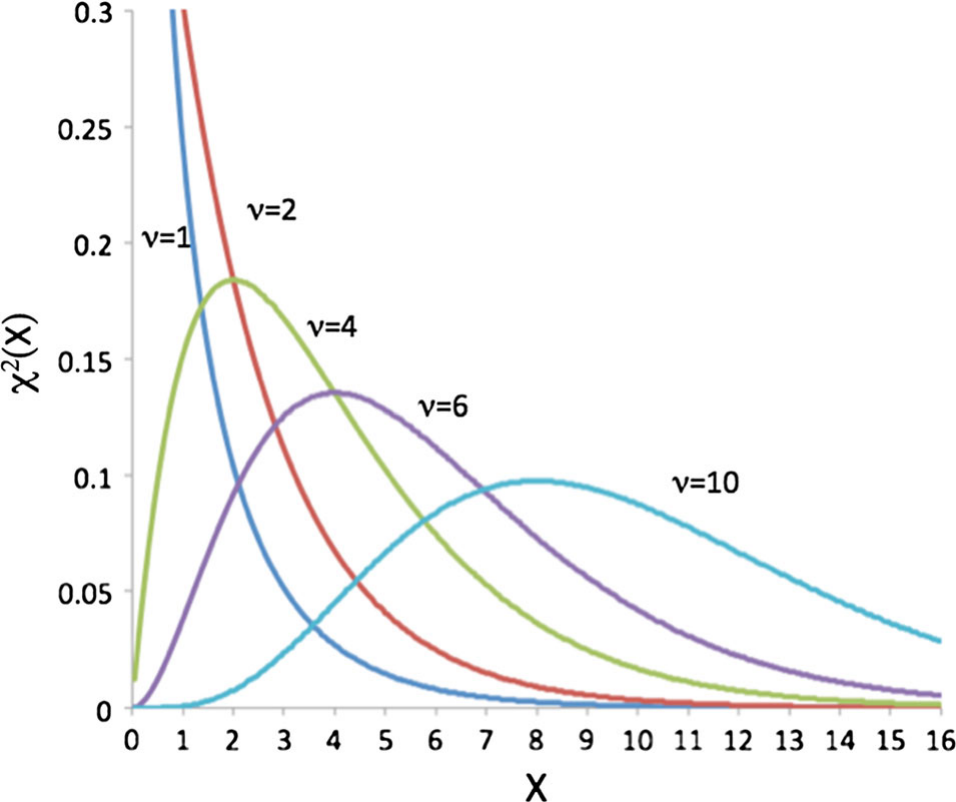
Illustrating the χ-squared function in Eq. 66, for ν = 1, 2, 4, 6 and 10. *Curves* are in order of their average from *left* to *right*. Note that the functions with *ν* > 2 peak at around *ν*-*2* but the average of all functions is actually ν

We return, now, to the problem of how to calculate the confidence limits of a variance-related quantity, for instance an *RMSE*. As was shown earlier, the standard deviation of a variance has a very simple expression, namely the variance multiplied by the square root of 2.0. However, as with many of the properties above, there is a natural boundary to the variance, i.e. since it is a squared quantity it cannot be less than zero. This means that the distribution of the variance about its expected value cannot be Gaussian. In the examples above this was tackled by a transformation of variables into a form in which the variance was once again Gaussian. Here it is easier to simply use the properties of the actual distribution, i.e. the sum of the squares of differences from a set of values. That distribution is known as the *Chi* *squared* distribution (χ-squared). Just like the Student *t*-function its shape depends on the sample number. Equation 66 gives the definition of the function and Fig. 9 provides some example distributions:

66 $$\chi (x,\upupsilon) = \frac{1}{{2^{{\frac{\nu }{2}}} \varGamma \left( {\frac{\upnu}{2}} \right)}}x^{{\frac{\upupsilon}{2} - 1}} e^{{ - \frac{x}{2}}}$$

The χ-squared function is the distribution of the sum of squares of random numbers chosen from a unit Gaussian (i.e. centered at zero with standard deviation of 1.0). The average of the square of a random number is just the standard deviation, i.e. here set to 1.0; so the average of the χ-squared function for *N* Gaussians is just *N*. When *N* is high it also resembles a Gaussian function, which is why the naïve application of the “variance of the variance” works well when *N* is large; see, for example the right most curve in Fig. 9.

The χ-squared function has many uses in statistics. It is used in assessing the quality of fit of an observed distribution to a theoretical prediction, as in the classic Chi squared test, in assessing if classification criteria are independent, in non-parametric tests such as Friedman’s Test for distinguishing ranked quantities, in the derivation of Fisher’s F-test (basically a ratio of two χ-squared functions), which can be used to see if the addition of parameters sufficiently improves a model. Here all we need are ranges for 95 % confidence limits. In the examples from the *Basics* section we had an RMSE of 2.0 kcal/mol for some affinity prediction based first on fifty samples and then on eight.

#### Example 1: Fifty samples

67 $$s_{50} = \frac{{\mathop \sum \nolimits_{i = 1}^{50} \left( {x_{i} - \bar{x}} \right)^{2} }}{49}$$

68 $$49s_{50} = \mathop \sum \limits_{i = 1}^{50} \left( {x_{i} - \bar{x}} \right)^{2}$$

i.e. the right hand side is equal to the sum of fifty randomly distributed square numbers. If these were drawn from a unit Gaussian the 95 % range of this sum would be from 32.36 to 71.42 (from table look-up of χ-squared values for the 95 % range for *N* = 50). Therefore we know:

69 $$32.36\sigma^{2} < 49s_{50} < 71.42\sigma^{2}$$

Here *σ* ^*2*^ provides the appropriate scaling since the numbers are not drawn from a unit Gaussian. This can be rearranged to give:

$$\frac{{49s_{50} }}{71.42} < \sigma^{2} < \frac{{49s_{50} }}{32.36}$$

i.e. substituting s_50_ = 2.0^2^ we get:

70 $$\frac{49 * 4.0}{71.42} < \sigma^{2} < \frac{49 * 4.0}{32.36}$$

Finally:

71 $$1.67 < \sigma < 2.46$$

These compare to the error bars from a Gaussian distribution of [1.54, 2.37], i.e. the error from the Gaussian-based Eq. 14 is slightly too conservative.

#### Example 2: Eight samples

By table lookup for the 95 % range for χ-squared for eight variables we obtain 2.18 and 17.56. Therefore:

72 $$\frac{7 * 4.0}{17.56} < \sigma^{2} < \frac{7 * 4.0}{2.18}$$

Leading to:

73 $$1.26 < \sigma < 3.58$$

Notice that the lower error bound is now much closer to the estimation of 2.0 because it is being “forced” away from the lower bound of 0.0, whereas the upper bound has moved up considerably.

The general formula for these error bounds is simply:

74 $$\sqrt {\frac{{(N - 1)\sigma^{2} }}{{\chi_{higher} }}} < \sigma < \sqrt {\frac{{(N - 1)\sigma^{2} }}{{\chi_{lower} }}}$$

Where the upper and lower χ bounds are from table lookup.

## Combining errors from different sources

### General formulae and examples

As we have seen in the case of the total error for AUC or Enrichment, there can be multiple sources of error that have to be combined to arrive at the total error. We have also seen that when we have a function of a random variable we can scale the expected contribution to the variance by the square of the rate of change of that function with respect to the noisy variable. These two observations can be joined to give a single statement as to how multiple sources of *independent* variability add to give the total variability. Given a function *X* such that:

75 $$\begin{aligned} &X\left( {x_{1} ,x_{2} ,x_{3} , \ldots ,x_{n} } \right) \hfill \\ &var(X) = \mathop \sum \limits_{i = 1}^{N} \left( {\frac{\partial X}{{\partial x_{i} }}} \right)^{2} var(x_{i} ) \hfill \\ \end{aligned}$$

A typical proof can be found in “Appendix 3” along with its extension to the case in which the variables are *not* independent:

76 $$var\left( X \right) = \mathop \sum \limits_{i = 1}^{N} \left( {\frac{\partial X}{{\partial x_{i} }}} \right)^{2} var\left( {x_{i} } \right) + \mathop \sum \limits_{i = 1}^{N} \mathop \sum \limits_{j \ne i}^{N} \left( {\frac{\partial X}{{\partial x_{i} }}} \right)\left( {\frac{\partial X}{{\partial x_{j} }}} \right)cov(x_{i} ,x_{j} )$$

Here the covariance of variables is as defined previously (Eq. 37).

To arrive at the total error we take the square root of the sum of the component variances, each weighted by the square of how quickly the composite variable changes with respect to the component. In the simplest case where X is a sum of equal terms:

77 $$var(X) = \mathop \sum \limits_{i = 1}^{N} var(x_{i} )$$

For example, if we want to know the variance of a sum of AUCs for a set of docking experiments, we simply add the variances. If we are estimating the binding energy of a ligand and have several *independent* components then the variance of the total energy is the sum of the variance of the parts.

The same formula is used for the *difference* between properties, i.e. if:

78 $$\begin{aligned} &X = x_{2} - x_{1} \hfill \\ &var\left( X \right) = var\left( {x_{1} } \right) + var\left( {x_{2} } \right) \hfill \\ \end{aligned}$$

In either case we are using the fact that:

79 $$\left( {\frac{\partial X}{{\partial x_{1} }}} \right)^{2} = \left( {\frac{\partial X}{{\partial x_{2} }}} \right)^{2} = 1$$

More generally, if the formula for X is:

80 $$X = \mathop \sum \limits_{i = 1}^{N} a_{i} x_{i}$$

Then:

81 $$var(X) = \mathop \sum \limits_{i = 1}^{N} a_{i}^{2} var(x_{i} )$$

Sometimes it is important to find the right function of X, the composite variable, in which to assess how errors can compound. Consider selectivity, i.e. the ratio of two binding affinities, *K*_*d*_.

82 $$S_{AB} = \frac{{K_{d}^{A} }}{{K_{d}^{B} }}$$

Suppose there is some natural error in the experimental binding of affinity such that *K*_*d*_ is potentially off by a factor of 10. The first thing we note is that we do not mean:

$$K_{d} = \left[ {\overline{{K_{d} }} - 10,\overline{{K_{d} }} + 10} \right]$$

We mean:

83 $$K_{d} = \left[ {0.1\overline{{K_{d} }} , 10\overline{{K_{d} }} } \right]$$

As such, the correct variable to express variances is the logarithm (base 10) of the binding constant:

84 $${ \log }(K_{d} ) = \left[ {\log \left( {\overline{{K_{d} }} } \right) - 1, \log \left( {\overline{{K_{d} }} } \right) + 1} \right]$$

Similarly,

85 $$\log \left( {S_{AB} } \right) = \log \left( {K_{d}^{A} } \right) - \log \left( {K_{d}^{B} } \right)$$

Now the variance of *log*(*S*) can be simply calculated:

86 $$\text{var} \left( {\log \left( {S_{AB} } \right)} \right) = \text{var} \left( {\log \left( {K_{d}^{A} } \right)} \right) + \text{var} \left( {\log \left( {K_{d}^{B} } \right)} \right) = 2 * \log (10) = 2$$

Therefore,

$$\log \left( {S_{AB} } \right) = \langle \log \left( {S_{AB} } \right)\rangle \pm t_{95\,\% } \sqrt 2$$

If we set the *t* statistic to 2.0 we get:

87 $$S_{AB} = \left[ {S_{AB} /673.6,673.6 * S_{AB} } \right]$$

Needless to say, this is a very wide range, and comes about, in part from the addition of errors. The problem of expected errors in selectivity estimation is analyzed in practical detail in [41].

In the above example the ratio was really a log-difference. In the case of the actual ratio of two quantities, perhaps the ratio of two permeability values, we have:

88 $$R = \frac{Y}{Z}$$

89 $$var(R) = \frac{1}{{Z^{2} }}var(Y) + \left( {\frac{{Y^{2} }}{{Z^{4} }}} \right)var(Z)$$

For instance, if a Caco2 permeability value of *Y* is 15.0 ± 2.0 * 10^−6^ cm/s and *Z* is 5.0 ± 1.0 * 10^−6^ cm/s, then the error on the ratio *R* is:

90 $$\begin{aligned} R = & 3 \pm t_{95\,\% } \sqrt {\frac{4}{25} + \frac{225}{625}} \\ R = & 3 \pm 0.72t_{95\,\% } \\ \end{aligned}$$

Another application to ratios concerns two commonly used measures of merit, Cohen’s “effect size”, *d*:

91 $$d = \frac{{\left\lceil {\bar{x}_{2} - \bar{x}_{1} } \right\rceil }}{\sigma }$$

And the coefficient of variation:

92 $$c_{v} = \frac{\sigma }{\mu }$$

Cohen’s *d* is more usually a measure of the difference between two properties, a topic explored in detail in the second article. Here we will consider that second value to be that of a “NULL” model, i.e. zero, in which case:

93 $$d = \frac{\mu }{\sigma }$$

I.e. the inverse of C_v_. Cohen’s *d* is used as a measure of importance of an effect, i.e. given the noisiness of a factor, how much does the effect stand out from that noise. Unlike the *t* statistic, it does not have a dependence on the sample size, which means it is an intrinsic property of the effect. It is equivalent to the “oomph” described by Ziliak and McCloskey [7]. The coefficient of variation is typically used as a measure of assay accuracy and requires an absolute scale for μ, the mean, e.g. a measure of permeability plus or minus 10 % would have a coefficient of variation of 0.1. It is also an intrinsic property, but intrinsic to the assay.

As we know the variance of a mean and also the variance of a variance we can apply the above formula for the variance of a ratio:

94 $$\begin{aligned} var(d) = & \left( {\frac{1}{{\sigma^{2}
}}\sigma^{2} + \sqrt 2 \left( {\frac{{\mu^{2} }}{{\sigma^{4} }}}
\right)\sigma^{2} } \right) \\ = & \left( {1 + \sqrt 2 \left(
{\frac{{\mu^{2} }}{{\sigma^{2} }}} \right)} \right) \\ = & \left(
{1 + \sqrt 2 d^{2} } \right) \\ SE(d) = &\; \sqrt {\frac{{1 +
\sqrt 2 d^{2} }}{N}} \\ \end{aligned}$$

95 $$\begin{aligned} var\left( {c_{V} } \right) = & \left(
{\frac{1}{{\mu^{2} }}\sqrt 2 \sigma^{2} + \left(
{\frac{{\sigma^{2} }}{{\mu^{4} }}} \right)\sigma^{2} } \right) \\
= & \left( {\sqrt 2 \left( {\frac{{\sigma^{2} }}{{\mu^{2} }}}
\right) + \left( {\frac{{\sigma^{4} }}{{\mu^{4} }}} \right)}
\right) \\ = &\; \frac{{\sigma^{4} }}{{\mu^{4} }}\left( {1 + \sqrt
2 \left( {\frac{{\mu^{2} }}{{\sigma^{2} }}} \right)} \right)
\\ = &\; c_{V}^{4} \left( {1 + \sqrt 2 d^{2} } \right) \\
SE\left( {c_{V} } \right) = &\; c_{V}^{2} \sqrt {\frac{{1 + \sqrt
2 d^{2} }}{N}} = c_{V}^{2} SE\left( d \right) \\
\end{aligned}$$

The question arises as to whether we need to worry about a covariance component, i.e. is:

$$cov(\mu ,\sigma ) = 0?$$

It is a remarkable fact that this is both a necessary *and* sufficient condition for normality! I.e. if the variance and mean are independent then the distribution is Gaussian, and vice versa [42].

### The general error formula

Often we will not know what the derivative of the composite is with respect to the component. For instance, perhaps we know that several factors are important but not exactly how. Variation in any of these parameters can give rise to variation in the effect we care about. In this case the general statement is typically in terms of the net and component errors,

96 $$Err_{total} = \sqrt {\mathop \sum \limits_{i = 1}^{m} Err_{i}^{2} }$$

This is how *error propagation* is presented in engineering texts. They simply combine the variance of each component via the error it introduces. Here the error is, in effect, the product of the standard deviation of each ‘factor’, multiplied by the sensitivity of the composite variable, i.e. the rate of change with respect to the component variable.

Take, for example, the error in the pKa estimation of a functional group. This might involve the error in the measurement of the pKa of a model compound, the expected error in moving from the model to the actual compound, the error in the estimation of the influence of an external potential from some distal group, etc. Each term is introduced, perhaps empirically, as adding its own contribution to the variance of the composite variable, in this case pKa. If the thermodynamics of a process are hard to measure but there are several “canonical” steps in the process, each step will add its own contribution to the total error. For example, solubility can be looked upon as a two-step process: sublimation from the crystal form and solvation of the free species into water (or other solvent). Each step has its own energy component and error. Reaction rates often involve several steps, each of which can be measured, perhaps, to good precision, however the total rate may be inaccurate due to accumulation of error.

What is important here is to remember that there may be multiple sources of error. If they can be estimated individually they can be combined in an appropriate manner, e.g. as in the general equations above.

### Estimating unknown contributions to error

The general formula can also be used in “reverse”. Suppose we have empirically measured the total error and believe we know all but one of the contributions to that error, *m*, then:

97 $$Err_{m} = \sqrt {Err_{observed}^{2} - \mathop \sum \limits_{i = 1}^{m - 1} Err_{i}^{2} }$$

To paraphrase Sherlock Holmes [43], when all other sources of error have been removed, what remains must be the missing error.

As an example, suppose we have a computational technique, say docking, and a test set of structures. We diligently apply our docking program to the test set, recording the range of performance statistics across this set. Now we give out both the test set and the software to the community to perform their own validations. To our surprise the results that are returned are not the same as ours but vary considerably depending on who used the program. For instance, it has been demonstrated that some programs with stochastic components behave quite differently on different platforms [6], or it could be because different program presets were used. Overall, the variance of performance is a reflection of user variability. If we know all other sources of error, for instance the limited number of systems, the limited number of actives and decoys and so on, then the remaining error is simply user variability.

Alternatively, we could look at our own evaluation in more detail. Some variability will arise because of the range of aptitude of the technique for different protein systems and some will arise because of the finite number of actives and decoys used for each system. As we can estimate the expected error for the latter, we can calculate a more accurate estimate of the intrinsic performance across different systems, i.e. we can estimate what our variability would have been if we had an infinite number of actives and decoys.

To examine this further, the equation for the total error in a property that is averaged over *N* systems is:

98 $$Err_{observed} = \sqrt {Err_{intrinsic }^{2} + \mathop \sum \limits_{i = 1}^{N} Err_{i}^{2} /\left( {N\left( {N - 1} \right)} \right)}$$

Here *Err*_*i*_ is the error in *i* ^th^ system. For example, suppose we were using the DUD dataset to evaluate a docking program. We would like to know what the error in our evaluation of the average AUC across all forty systems might be. To do this we would evaluate the standard error as:

99 $$SE_{DUD} = \sqrt {\frac{{\sigma_{AUC}^{2} }}{N}} = \sqrt {\frac{{\mathop \sum \nolimits_{i = 1}^{N} \left( {AUC_{i} - \overline{AUC} } \right)^{2} }}{N(N - 1)}}$$

Where *N* = 40. Now, each system has a finite number of actives and decoys and so each *AUC*_*i*_ in the above equation has some error, which will add to the total error. It would be nice if we knew the standard error as if there were no additional error from each system, i.e. if all that was left was the true variance between systems. But we know the error of each system is independent of every other system and so we can rewrite the equation for the standard error as:

100 $$\begin{aligned} SE_{DUD} = & \sqrt {\frac{{\mathop \sum \nolimits_{i = 1}^{i = N} \left( {AUC_{i}^{\infty } + Err_{i} - \overline{AUC} } \right)^{2} }}{N(N - 1)}} \\ SE_{DUD} = & \sqrt {\left( {SE_{DUD}^{\infty } } \right)^{2} + \frac{{\mathop \sum \nolimits_{i = 1}^{N} \left( {Err_{i} } \right)^{2} }}{N(N - 1)}} \\ \end{aligned}$$

Here the infinity symbol is used to imply the value we would expect if there were an infinite number of actives and decoys for each system, whereas *Err*_*i*_ is the error in the *i* ^th^ system because their number is finite. Therefore:

101 $$SE_{DUD}^{\infty } = \sqrt {\left( {SE_{DUD} } \right)^{2} - \frac{{\mathop \sum \nolimits_{i = 1}^{i = N} \left( {Err_{i} } \right)^{2} }}{N(N - 1)}}$$

I.e. we can estimate what the “true” standard error might be.

Here is an example for a virtual screening program using DUD as its dataset and AUC as the performance metric. The average AUC was 0.75.

102a $$\begin{aligned} &\sigma_{DUD, Observed} = 0.136 \hfill \\ &SE_{DUD} = \frac{0.136}{{\sqrt {40} }} = 0.022 \hfill \\ \end{aligned}$$

$$\sigma_{system}^{2} = \frac{{\mathop \sum \nolimits_{i = 1}^{i = N} \left( {Err_{i} } \right)^{2} }}{N - 1} = 0.0038$$

Here the *Err*_*i*_ are calculated using the Hanley formula assuming an excess of decoys to actives. Therefore:

102b $$SE_{DUD}^{\infty } = \sqrt {\left( {0.022} \right)^{2} - (0.0038/40)} = 0.0165$$

I.e. we have improved the error bound in Eq. 102a by removing the effect of the expected error over the systems within DUD.

### Assessing adding noisy systems to test sets

Now, suppose we are considering adding a new system to our set of test systems, but we know this new system is going to be noisy, i.e. perhaps there are relatively few active compounds for this protein to use as exemplars. Is it worth adding the new system? If we add it there would be more systems, so the average performance should now be more accurate. However, we know that improvement in the mean comes slowly, i.e. because of the *√N* effect. Will the noise in this new system overcome the advantages of *N* increasing to *N* + 1? Is more data actually better? As derived in “Appendix 5”, there is a simple criterion for this:

103 $${\sigma_{intrinsic}}^{2} + 2\sigma_{system}^{2} > Err_{N + 1}^{2}$$

Where:

104 $${\sigma_{intrinsic}}^{2} = N * \left( {SE_{DUD}^{\infty } } \right)^{2}$$

And:

105 $$\sigma_{system}^{2} = \frac{{\mathop \sum \nolimits_{i = 1}^{i = N} \left( {Err_{i} } \right)^{2} }}{N - 1}$$

In situations such as docking evaluations the first term tends to dominate, i.e. the variance between systems is much larger than the noise within any one system. As such this condition can be approximated with:

106 $$\sigma_{intrinsic}^{2} > Err_{N + 1}^{2}$$

This makes intuitive sense, i.e. if the intrinsic noise is larger than that of the system being added then that system can only help.

Notice that this is not “cherry-picking”. Cherry-picking is when systems are removed from the tally to make the average AUC look better. What we are doing is looking to see if we can reduce the *noise* in the estimation of the mean AUC. Suppose we return to the DUD dataset and the actual data from a docking evaluation. The smallest number of actives in DUD is for the catechol methyl-transferase protein, for which there are only eight. The expected system error for this protein, given that the virtual screening program achieved an AUC of 0.63, is:

107 $$Err_{N + 1}^{2} \approx \frac{{AUC^{2} \left( {1 - AUC} \right)}}{{\left( {1 + AUC} \right)N_{actives} }} = \frac{{0.63^{2} * 0.37}}{{\left( {1 + 0.63} \right) * 8}} = 0.0113$$

The intrinsic variance of the system, assuming we started with 39 systems, i.e. had not included catechol methyl-transferase, is:

108 $$\sigma_{intrinsic}^{2} = N * \left( {SE_{DUD}^{\infty } } \right)^{2} = 39 * \left( {0.0165} \right)^{2} = 0.0106$$

The system variance was:

109 $$\sigma_{system}^{2} = \frac{{\mathop \sum \nolimits_{i = 1}^{i = N} \left( {Err_{i} } \right)^{2} }}{N - 1} = 0.0017$$

Therefore:

110 $${\sigma_{intrinsic}}^{2} + 2\sigma_{system}^{2} = 0.0123 > Err_{N + 1}^{2} = 0.0113$$

As such the decision to include the likely noisy system of catechol methyl-transferase was justified, but it is not adding much signal! It should be noted that our derivation required the AUC for this system as a part of the estimation of the expected error. This is a limitation of the method, which could be abrogated by using an expected AUC, e.g. the average over all systems. This would have made little difference to the conclusion.

Finally, we have assumed the noise in the system as coming from the intrinsic variance of the method, i.e. if we had an infinite number of actives and decoys, plus the expected variance from the systems. There could be other sources of noise, for instance we mentioned above the potential contribution from different users applying the program. These terms would become a part of what we would see as the intrinsic variance.

### Variance-weighted averages with examples

The second major consideration for modelers combining information is different measurements of the same thing, i.e. perhaps we measure a property *N* different ways. This is different from calculating an average metric over *N* systems. In the first case the *N* measurements, in the limit of perfect accuracy, are all the same. In the second, in the limit of perfect accuracy we expect an average over different numbers. We are considering cases such as when we want to combine several different *LogP* values, each measured using a different experimental technique. Here the underlying value is the same, it is the inaccuracy of experiments that leads to different values. The difference is important because not only is the formula for the combined error different, but that for the *mean* is different! In fact, the formula for the expected mean that will give the lowest, unbiased, expected root-mean square error is:

111 $$\bar{X} = \frac{{\mathop \sum \nolimits_{i = 1}^{N} X_{i} /Err_{i}^{2} }}{{\mathop \sum \nolimits_{i = 1}^{N} 1/Err_{i}^{2} }}$$

I.e. the most accurate mean we can obtain is a weighted average, where the weights are the reciprocals of the error (squared) for each measurement. Measurements that are inaccurate (larger errors) are down-weighted compared to those that are more accurate. If one measurement is very accurate compared to the others it dominates the sum.

One way to clarify when this formula should be used is to ask what would happen if one measurement was exceedingly accurate. In the case of combining *different* systems this would mean that the value associated with this *one* system would dominate the average—clearly not what we would want. However, if we are presented with several estimates of a *LogP* we would clearly be satisfied if one measurement was very accurate and would intuitively use it rather than combine it with other, less accurate values.

The formula for the expected error for combining measurements is just the harmonic average of the squares of the individual errors:

112 $$Err_{total}^{2} = \frac{1}{{\mathop \sum \nolimits_{i = 1}^{N} 1/Err_{i}^{2} }}$$

Hence, errors add but only via the reciprocal of the sum of their reciprocals. The effect, then, is that a single small error dominates the denominator and hence the total error. If errors are all roughly equal we retrieve the expected observation that the total error goes down by √*N*.

It should be noted that all terms in the denominator add, i.e. *all* measurements reduce the total error, even the noisy ones. It does not matter how bad a measurement is as long as we know *how* bad, i.e. the important caveat here is to actually know the error of a particular measurement. A more typical situation is that a computational chemist is presented with a set of measurements without associated error bars and then has to combine all values. In this situation more data can be worse. We illustrate this by considering the following situation. Suppose we have three measurements of a pKa as in Table 2 with associated errors shown.

**Table 2:** Table showing example pKa values for three measurements with associated SD for each experimental measurement

	pKa1	pKa2	pKa3
Value	4.2	4.4	4.9
SD	0.2	0.2	0.5

We consider three cases:

#### Case 1

Use all three measurements with the associated standard errors.

$$pKa = \frac{{{\raise0.7ex\hbox{${4.2}$} \!\mathord{\left/ {\vphantom {{4.2} {0.2^{2} + }}}\right.\kern-0pt} \!\lower0.7ex\hbox{${0.2^{2} + }$}}{\raise0.7ex\hbox{${4.4}$} \!\mathord{\left/ {\vphantom {{4.4} {0.2^{2} + }}}\right.\kern-0pt} \!\lower0.7ex\hbox{${0.2^{2} + }$}}{\raise0.7ex\hbox{${4.9}$} \!\mathord{\left/ {\vphantom {{4.9} {0.5^{2} }}}\right.\kern-0pt} \!\lower0.7ex\hbox{${0.5^{2} }$}}}}{{{\raise0.7ex\hbox{$1$} \!\mathord{\left/ {\vphantom {1 {0.2^{2} + }}}\right.\kern-0pt} \!\lower0.7ex\hbox{${0.2^{2} + }$}}{\raise0.7ex\hbox{$1$} \!\mathord{\left/ {\vphantom {1 {0.2^{2} + }}}\right.\kern-0pt} \!\lower0.7ex\hbox{${0.2^{2} + }$}}{\raise0.7ex\hbox{$1$} \!\mathord{\left/ {\vphantom {1 {0.5^{2} }}}\right.\kern-0pt} \!\lower0.7ex\hbox{${0.5^{2} }$}}}} = 4.34$$

$$SE = \sqrt {\frac{1}{{{\raise0.7ex\hbox{$1$} \!\mathord{\left/ {\vphantom {1 {0.2^{2} + }}}\right.\kern-0pt} \!\lower0.7ex\hbox{${0.2^{2} + }$}}{\raise0.7ex\hbox{$1$} \!\mathord{\left/ {\vphantom {1 {0.2^{2} + }}}\right.\kern-0pt} \!\lower0.7ex\hbox{${0.2^{2} + }$}}{\raise0.7ex\hbox{$1$} \!\mathord{\left/ {\vphantom {1 {0.5^{2} }}}\right.\kern-0pt} \!\lower0.7ex\hbox{${0.5^{2} }$}}}}} = 0.13$$

#### Case 2

Use all three measurements but assume the standard error of the third measurement is the same as that of the other two measurements.

$$pKa = \frac{4.2 + 4.4 + 4.9}{3} = 4.50$$

$$SE = \sqrt {\frac{{0.2^{2} }}{3}} = 0.12$$

#### Case 3

Decide the third measurement is unreliable and only use the first two measurements.

$$pKa = \frac{4.2 + 4.4}{2} = 4.30$$

$$SE = \sqrt {\frac{{0.2^{2} }}{2}} = 0.14$$

The most accurate result is from Case 1 with the correct variance weighting of data. Adding the third result without taking into account its expected error actually made things worse compared to leaving it out entirely. This illustrates the importance in knowing the accuracy of independent measurements. In addition, it can be used to assess the potential consequences of including a measurement of unknown accuracy.

This leads into the area of outlier removal that is beyond the scope of this article, but the concept is straightforward. Suppose we are suspicious of the third measurement, and of its uncertainty estimate. Using just the first two values we obtain an estimate of 4.30, plus an uncertainty of 0.14. This makes the third value of 4.9 appear unlikely unless it has a large variance. Pierce’s criterion [44] is to calculate the likelihood of all three observations, compared to the likelihood of two good measurements and one being in error. It is also similar in spirit to the discussion above as to whether adding a new, noisy system to a set improves or degrades the expected accuracy.

The variance-weighted formula is typically used in the field of meta-analysis, i.e. where an effect size is estimated by combining different studies of different inherent accuracy. However, there is no reason it cannot also be applied to problems in computational chemistry when results of widely different provenance and accuracy are involved.

### Weighted averages of variances

Sometimes the average we want is of the same property, i.e. as in the preceding example, but where we assign weights for some other reason to each property, i.e.

113 $$\langle x\rangle = \frac{{\mathop \sum \nolimits_{i = 1}^{N} W_{i} x_{i} }}{{\mathop \sum \nolimits_{i = 1}^{N} W_{i} }}$$

In this case an unbiased estimator of the standard deviation is:

114 $$\sigma^{2} = \frac{{\mathop \sum \nolimits_{i = 1}^{N} W_{i} }}{{\left( {\left( {\mathop \sum \nolimits_{i = 1}^{N} W_{i} } \right)^{2} - \mathop \sum \nolimits_{i = 1}^{N} W_{i}^{2} } \right)}}\mathop \sum \limits_{i = 1}^{N} W_{i} \left( {x_{i} - \langle x\rangle } \right)^{2}$$

Here the sum is exactly what one might expect, i.e. weighted sum of the deviations from the average, but the prefactor represents the bias-correction. In the case all weights are equal this prefactor becomes (1/*N* − 1) as expected. See “Appendix 1”, part (ii), for a proof of this form.

## Bootstrapping error bars

### Introduction

In recent years computing power has made it possible to estimate many statistical quantities without the need for analytic formulae. Put simply, the data at hand is resampled “with replacement” and each time the metric of interest is recalculated. The distribution of this set of recalculated numbers is then used to derive statistics of interest. The phrase “with replacement” just means that if you start with *N* observations, the new “random” set of *N* observations can contain (almost certainly will contain) repeated instances. E.g. if your dataset is {1, 4, 3, 2, 5} a bootstrapped sample might be {1, 1, 5, 3, 3}, i.e. same number of data points but drawn randomly from the original. As a rule of thumb, about one quarter of the data points will be used more than once. To get a 95 % confidence limit from bootstrapping you observe the range around the mean that contains 95 % of the resampled quantity of interest. Because our bounds are drawn from the calculated distribution they cannot exceed ‘natural’ limits, e.g. [0,1] for probabilities. Neither do we have to worry about small sample size effects, or effective degrees of freedom. No mathematics required! Just resample many times (typically thousands) until the statistics you desire seem stable. As computational time is these days cheap, this is feasible for nearly any application in the field of molecular simulation.

It can be tempting to assume that bootstrapping is all that is ever needed, but this is incorrect. An obvious obstacle is if the primary data is not available, or difficult to extract from its deposited form, e.g. embedded in a PDF file. It is surprising how often journals allow the deposition of hard-to-use data to count as ‘submission of data’. One wonders how far structural biology would have got if PDB files were only available as PDF files! With classical statistics a researcher can come up with on-the-fly estimate of confidence limits of a proposed finding. Such checks with reality are often useful at scientific meetings.

Having an analytic formula allows a scientist to think about the character of the error—e.g. what the dominant terms will be, how they will behave with respect to size. This should be a natural part of how scientists think about experiments and can get lost if everything is just simulated. When Schrödinger was presented with the result of an early computer-derived numerical solution to his famous equation he is supposed to have commented, “I know it (the computer) understands the answer; however, I’d like to understand it too”. Sometimes a result is all you need and at other times an understanding of the result is more important.

### Limitations

There are times when bootstrapping is not appropriate. Some of these circumstances are germane to computational chemistry:

(i) Bootstrapping may be problematic for calculating the *mode* of a set of observations, i.e. the most common occurrence. As you are introducing multiple copies of observations you can end up measuring how often you oversample a single observation [45].
(ii) Calculating the *maximum* or *minimum* of a function is also not natural for this procedure. Any resampling that leaves out the maximum or minimum can only underestimate these extrema, i.e. bootstrapping cannot help but average to a lower value than it should. This has relevance in the calculation of enrichment in virtual screening when the percent of inactives screened is so small essentially you are measuring the extreme values of the scores for actives.
(iii) A significant limitation of bootstrapping is the calculation of correlation coefficients. This makes intuitive sense from the character of a correlation. If we replace one of our data points with a duplicate of another then the correlation is likely (but not guaranteed) to increase, meaning that the average correlation of a sampled population may appear higher than the true correlation. Note this is *not* true for the distribution of the slope from linear regression, which is normally distributed.
(iv) Confidence limits far from the mean. The problem here is one of sampling. To get reliable confidence limits we need a sufficient number of examples that lie outside of these limits. This may be difficult or even impossible from resampling. “Difficult” because the number of resamplings may become prohibitive in order to see enough rare events. “Impossible” because if the sample set is small even the exhaustive evaluation of all possible samplings may not sample the extrema possible for this data set. E.g. imagine calculating the mean of the small set from the introduction to this section; no resamplings can give an average less than 1 or greater than 5, probability *p* = (0.2)^5^ = 0.00032 for each. Therefore, a significance level of 0.0001 can never be established.

There are clever ways around all these problems. For instance, you can combine the parametric approach (i.e. classical statistics) with bootstrapping, i.e. estimating the best parametric form with resampled data. There is the bias-corrected bootstrap method and the bias-corrected and accelerated method [46] that address some of the issues raised above in (i–iv) concerning the bias that can arise in bootstrapping. There is also "smooth" bootstrapping where a small amount of noise is added to each resampled observation, Bayesian bootstrapping where weights on the original data are resampled to give different posterior distributions, “wild” bootstrapping, block bootstrapping etc. In other words, although bootstrapping can address most issues *if care is taken*, it is no different from classical statistics in that experience is required to choose the right approach. Effron, who is largely responsible for the modern bootstrapping method puts it well [47]:

"A good way to think of bootstrap intervals is as a cautious improvement over standard intervals, using large amounts of computation to overcome certain deficiencies of the standard methods, for example its lack of transformation invariance. The bootstrap is not intended to be a substitute for precise parametric results but rather a way to reasonably proceed when such results are unavailable." (p. 295)

### Advantages

However, there are real advantages. As bootstrapping is *non*-*parametric*, it can be very appropriate if the actual distribution is unusual. As an example, let’s briefly reconsider the very concept of the confidence interval. Traditional methods give such a range as half of the confidence interval above the mean and half below. As we have seen the upper and lower ranges don’t have to be symmetric. Useful tricks such the Fisher transform can sometimes get us estimates of ranges about the mean anyway, but perhaps the distribution of the value we are interested in looks nothing like a Gaussian, or a Student-t function, or any other parametric form. Then bootstrapping comes into its own. A good example of this type of distribution can be seen in Shalizi [48] where, in a very readable article, he looks at the day-to-day returns of the stock market.

## Conclusions

We have presented here the first part of a description of how confidence intervals for quantities frequently found in computational chemistry may be assessed. Issues such as asymmetrical error bars, small sample sizes and combining sources of error have been addressed, and a survey of analytic results, some old and some new. The importance of the latter as a method of thinking about the expected error has been emphasized, although certainly modern methods such as bootstrapping do offer alternatives to “back of the envelope” estimates. It would be impossible for such an article to cover all the clever formulae and techniques applicable to modeling even in just classical, parametric statistics; both modeling and statistics are too large a subject matter for that. Nor was it possible to cover much in the way of applicable non-parametric statistics, even though they are an attractive alternative when data is not normally distributed or where more robust measures are required. Not that those classical techniques cannot be made robust, but there was little room to describe these techniques either! Most regretfully, more could not be introduced concerning the Bayesian formalism, a path, which once started upon is hard to turn from, so general and powerful is the approach. However, there are many excellent textbooks that cover these and other aspects of statistics that may be useful to a computer modeler [49–52].

The follow-on paper to this will address the comparison of two or more quantities with associated error estimates. This involves the calculation of “mutual” error bars, i.e. confidence internals on the difference in properties. Here attention must be paid to the covariance aspect of variation, i.e. if the noisiness we hope to quantify for one property is correlated with the noise in another we have to exercise care in how this mutual difference is assessed. In our opinion this second paper contains more novel and research oriented material, simply because of the dearth of material even in the statistical literature on some topics, such as the effects of correlation on Pearson’s *r*-value. The effects of correlation can be subtle and it is hoped the results presented will prevent others from making the same mistakes as the author has made when first presented with the issues that arise.

In terms of affecting the standards of statistics displayed in the literature there is only so much that can be done without the coordinated efforts of journals and scientists. It requires standards to both be set and adhered to if computational chemistry is to improve in this way. Fortunately there seems to be a more general consensus that statistics should be taken more seriously in the sciences [53, 54], perhaps as a result of the number of retracted studies or papers illustrating how often reports are later contradicted by more thorough studies [55]. There should be no illusions that statistical standards will solve the many problems of our field. Our datasets are usually poorly constructed and limited in extent. Problems are selected because they give the results we want, not to reflect an accurate sampling of real world application. Poor or negative results are not published [56]. In addition, there is nothing to stop the inappropriate use of statistics, whether inadvertent or with intent to mislead. Many regrettable habits will not be affected.

It is not this author’s intent or purpose to set or even suggest such standards. Rather, this paper and the one that follows are attempts to communicate the potential richness and productive character of statistics as applied to our very empirical field. As an aid to the adoption of the methods presented here it is intended to develop a website, caddstat.eyesopen.com that will provide on-line forms for statistical calculation relevant to computational chemistry. This site will be described in a subsequent publication.

For any field to progress it must be able to assess its current state, and statistics provides the tools for that assessment. Used honestly and consistently statistical techniques allow a realistic perspective to emerge of our field's problems and successes. As drug discovery becomes harder and more expensive, it is ever more important that the application of computation methods actually deliver their original promise of speeding and improving pharmaceutical design. A more statistically informed field should be a part of that future.

## Appendix 1: Unbiased estimates of variance

### Equally weighted average

We have a quantity *x* and we want to know its standard deviation. If we *know* the average of this property, μ, then the formula for this is:

115 $$\sigma^{2} = \frac{1}{N}\mathop \sum \limits_{i = 1}^{N} (x_{i} - \mu )^{2}$$

However, if we have to estimate the average, μ_0_, i.e. by using the mean of the observed *x* value, then the formula is:

116 $$\sigma^{2} = \frac{1}{N - 1}\mathop \sum \limits_{i = 1}^{N} (x_{i} - \mu_{0} )^{2}$$

Where,

117 $$\mu_{0} = \frac{1}{N}\mathop \sum \limits_{i = 1}^{N} x_{i}$$

This is called the ‘sample’ standard deviation, because *N* is finite and because we do not have the true mean, i.e. there is one less degree of freedom than there seems.

The following reasoning can be used to arrive at this unbiased estimator: Assume that the true average, *μ,* is zero. There is no loss of generality, here, as we can always shift each *x* by *μ* to make this so. This does not mean that μ_0_ is also zero, just the actual mean. In this case, as we have set the true mean to zero:

118 $$\sigma^{2} = \frac{1}{N}\mathop \sum \limits_{i = 1}^{N} x_{i}^{2} = \langle x^{2} \rangle$$

Consider the formula in this case for the unbiased estimator:

119 $$\begin{aligned} \sigma^{2} & = \frac{1}{N - 1}\mathop \sum \limits_{i = 1}^{N} (x_{i} - \mu_{0} )^{2} \\ \sigma^{2} & = \frac{1}{N - 1}\mathop \sum \limits_{i = 1}^{N} \left( {x_{i} - \frac{1}{N}\mathop \sum \limits_{i = 1}^{N} x_{i} } \right)^{2} \\ \sigma^{2} & = \frac{1}{N - 1}\mathop \sum \limits_{i = 1}^{N} \left( {\frac{N - 1}{N}x_{i} - \frac{1}{N}\mathop \sum \limits_{j \ne i}^{N} x_{j} } \right)^{2} \\ \sigma^{2} & = \frac{1}{N - 1}\mathop \sum \limits_{i = 1}^{N} \left[ {\left( {\frac{N - 1}{N}} \right)^{2} x_{i}^{2} - \frac{N(N - 1)}{{N^{2} }}\mathop \sum \limits_{j \ne i}^{N} x_{i} x_{j} + \left( {\frac{1}{N}\mathop \sum \limits_{j \ne i}^{N} x_{j} } \right)^{2} } \right] \\ \end{aligned}$$

Now, the key observation is that quantities such as (*x*_*i*_ * *x*_*j*_) must average to zero because we have assumed the true mean is equal to zero, and we assume the *x*_*i*_ are independent. Given this, the above simplifies to:

120 $$\begin{aligned} \sigma^{2} & = \frac{1}{N - 1}\mathop \sum \limits_{i = 1}^{N} \left[ {\left( {\frac{N - 1}{N}} \right)^{2} x_{i}^{2} + \left( \frac{1}{N} \right)^{2} \mathop \sum \limits_{j \ne i}^{N} x_{j}^{2} } \right] \\ \sigma^{2} & = \frac{1}{N - 1}\mathop \sum \limits_{i = 1}^{N} \left[ {\left( {\frac{N - 1}{N}} \right)^{2} x_{i}^{2} + \frac{N - 1}{{N^{2} }}x_{i}^{2} } \right] \\ \sigma^{2} & = \frac{1}{N - 1}\mathop \sum \limits_{i = 1}^{N} \left[ {\frac{{\left( {N - 1} \right)^{2} + (N - 1)}}{{N^{2} }}x_{i}^{2} } \right] \\ \sigma^{2} & = \frac{1}{N - 1}\mathop \sum \limits_{i = 1}^{N} \left[ {\frac{(N - 1)}{N}x_{i}^{2} } \right] \\ \sigma^{2} & = \frac{1}{N}\mathop \sum \limits_{i = 1}^{N} x_{i}^{2} = \frac{1}{N}\mathop \sum \limits_{i = 1}^{N} \left( {x_{i} - 0} \right)^{2} \\ \end{aligned}$$

Which is the correct formula for the standard deviation when the true mean is equal to zero. Therefore, the 1/(N − 1) term shifts us from the variance calculated using the assumed mean to that of the variance with the actual mean.

### Weighted average

Now consider a weighted average. Consider the quantity:

121 $$s^{2} = \mathop \sum \limits_{i = 1}^{N} w_{i} \left( {x_{i} - \mu_{0} } \right)^{2}$$

Where the *w*_*i*_ are weights. We assume, for the moment that the weights add up to one. Then, we have:

122 $$\begin{aligned} s^{2} & = \mathop \sum \limits_{i = 1}^{N} w_{i} x_{i}^{2} - 2\mu_{0} \mathop \sum \limits_{i = 1}^{N} w_{i} x_{i} + \mu_{0}^{2} \mathop \sum \limits_{i = 1}^{N} w_{i} \\ s^{2} & = \mathop \sum \limits_{i = 1}^{N} w_{i} x_{i}^{2} - \mu_{0}^{2} \\ s^{2} & = \mathop \sum \limits_{i = 1}^{N} w_{i} x_{i}^{2} - \mathop \sum \limits_{i = 1}^{N} w_{i}^{2} x_{i}^{2} \\ \end{aligned}$$

Where we have used the same trick as above, i.e. that (*x*_*i*_ * *x*_*j*_) must average to zero. The final step is to realize that all the *x*_*i*_^2^ terms in the last equation are independent and are assumed to have the same variance. So:

123 $$\begin{aligned} s^{2} & = \langle x^{2} \rangle \mathop \sum \limits_{i = 1}^{N} w_{i} - \langle x^{2} \rangle \mathop \sum \limits_{i = 1}^{N} w_{i}^{2} \\ s^{2} & = \langle x^{2} \rangle \left( {1 - \mathop \sum \limits_{i = 1}^{N} w_{i}^{2} } \right) \\ \end{aligned}$$

Therefore:

124 $$\sigma^{2} = \langle x^{2} \rangle = \frac{1}{{\left( {1 - \mathop \sum \nolimits_{i = 1}^{N} w_{i}^{2} } \right)}}\mathop \sum \limits_{i = 1}^{N} w_{i} \left( {x_{i} - \mu_{0} } \right)^{2}$$

If the original weights did not sum to one, we would have normalized them thus:

125 $$w_{i} = \frac{{W_{i} }}{{\mathop \sum \nolimits_{i = 1}^{N} W_{i} }}$$

As such, transforming back to the original weights gives:

126 $$\begin{aligned} \sigma^{2} & = \frac{1}{{\left( {1 - \mathop \sum \nolimits_{i = 1}^{N} W_{i}^{2} /\left( {\mathop \sum \nolimits_{i = 1}^{N} W_{i} } \right)^{2} } \right)}}\mathop \sum \limits_{i = 1}^{N} \frac{{W_{i} }}{{\mathop \sum \nolimits_{i = 1}^{N} W_{i} }}\left( {x_{i} - \mu_{0} } \right)^{2} \\ \sigma^{2} & = \frac{{\left( {\mathop \sum \nolimits_{i = 1}^{N} W_{i} } \right)^{2} }}{{\left( {\left( {\mathop \sum \nolimits_{i = 1}^{N} W_{i} } \right)^{2} - \mathop \sum \nolimits_{i = 1}^{N} W_{i}^{2} } \right)}}\frac{1}{{\mathop \sum \nolimits_{i = 1}^{N} W_{i} }}\mathop \sum \limits_{i = 1}^{N} W_{i} \left( {x_{i} - \mu_{0} } \right)^{2} \\ \end{aligned}$$

Equation 126 is the desired result.

## Appendix 2: The variance of the variance

We want to know the expected error in the standard deviation or, equivalently, the variance. Therefore, the quantity we want is:

127 $$var\left( {var(x)} \right) = \frac{{\mathop \sum \nolimits_{i = 1}^{N} \left( {x^{2} - \overline{{x^{2} }} } \right)^{2} }}{N - 1}$$

As in the calculation of the sample variance, *N* − 1 is included to correct for the bias introduced by using the sample estimate of the average value of *x* ^2^. This can be expanded to:

128 $$var\left( {var(x)} \right) = \frac{{\mathop \sum \nolimits_{i = 1}^{N} x^{4} - \left( {\overline{{x^{2} }} } \right)^{2} }}{N - 1}$$

Now, if the errors are normal, then by definition:

129 $$\overline{{x^{2} }} = (2\pi \sigma^{2} )^{ - 1/2} \mathop \smallint \limits_{ - \infty }^{ + \infty } x^{2} e^{{ - \frac{{x^{2} }}{{2\sigma^{2} }}}} = \sigma^{2}$$

And:

130 $$\overline{{x^{4} }} = (2\pi \sigma^{2} )^{ - 1/2} \mathop \smallint \limits_{ - \infty }^{ + \infty } x^{4} e^{{ - \frac{{x^{2} }}{{2\sigma^{2} }}}} = 3\sigma^{4}$$

Therefore:

131 $$\begin{aligned} &var\left( {var(x)} \right) = 3\sigma^{4} - \left( {\sigma^{2} } \right)^{2} \hfill \\ &var\left( {var(x)} \right) = 2\sigma^{4} \hfill \\ \end{aligned}$$

Hence, the standard deviation of the variance is:

132 $$SD\left( {var(x)} \right) = \sqrt 2 \sigma^{2}$$

And the standard error is:

133 $$SE\left( {var(x)} \right) = \sqrt 2 \sigma^{2} /\sqrt {N - 1}$$

## Appendix 3: Transforming Variance

### A single variable

Suppose we have a function *H* of variable *x*, and we know the standard deviation of *x*. What is the variance of *H*? By definition we have

134 $$Var\left( {H(x)} \right) = \int {\left( {H(x) - \overline{H(x)} } \right)^{2} p(x) \cdot dx}$$

Where *p*(*x*) is the probability distribution function for *x*. So to calculate the variance we also need to know the average value of *H*. This we can obtain from:

135 $$\overline{H\left( x \right)} = \int {H(x)p(x)dx}$$

Suppose we make a Taylor expansion of *H*(*x*) around the mean of *x* inside of the integral:

136 $$\overline{H(x)} = \int {\left( {H(\bar{x}) + \left. {\frac{\partial H}{\partial x}} \right|_{{x = \bar{x}}} \left( {x - \bar{x}} \right) + \frac{1}{2}\left( {\left. {\frac{{\partial^{2} H}}{{\partial x^{2} }}} \right|_{{x = \bar{x}}} } \right)\left( {x - \bar{x}} \right)^{2} + \cdots } \right)} p(x)dx$$

The integral of the second term is zero, leaving:

137 $$\begin{aligned} &\overline{H(x)} = H\left( {\bar{x}} \right) + \frac{1}{2}\left( {\left. {\frac{{\partial^{2} H}}{{\partial x^{2} }}} \right|_{{x = \bar{x}}} } \right)\int {x - \bar{x}}^{2} p(x)dx + higher\;order\;terms \hfill \\ &\overline{H(x)} = H\left( {\bar{x}} \right) + \frac{1}{2}\left( {\left. {\frac{{\partial^{2} H}}{{\partial x^{2} }}} \right|_{{x = \bar{x}}} } \right)\sigma_{x}^{2} + higher\;order\;terms \hfill \\ \end{aligned}$$

Returning to Eq. 134 but expanding *H*(*x*) around the mean of *x* in a similar way, and substituting the first two terms on the right hand side of Eq. 137 for the average value of *H* we obtain:

138 $$\begin{aligned} Var\left( {H(x)} \right) & = \int {\left( {H(\bar{x}) + \left( {\left. {\frac{\partial H}{\partial x}} \right|_{{x = \bar{x}}} } \right)\left( {x - \bar{x}} \right) + \frac{1}{2}\left( {\left. {\frac{{\partial^{2} H}}{{\partial x^{2} }}} \right|_{{x = \bar{x}}} } \right)\left( {x - \bar{x}} \right)^{2} + higher\;order\;terms - H(\bar{x}) - \frac{1}{2}\left( {\left. {\frac{{\partial^{2} H}}{{\partial x^{2} }}} \right|_{{x = \bar{x}}} } \right)\sigma_{x}^{2} } \right)}^{2} p(x)dx \\ Var\left( {H(x)} \right) & = \int {\left( {\left( {\left. {\frac{\partial H}{\partial x}} \right|_{{x = \bar{x}}} } \right)\left( {x - \bar{x}} \right) + \frac{1}{2}\left( {\left. {\frac{{\partial^{2} H}}{{\partial x^{2} }}} \right|_{{x = \bar{x}}} } \right)\left( {\left( {x - \bar{x}} \right)^{2} - \sigma_{x}^{2} } \right) + higher\;order\;terms} \right)}^{2} p(x)dx \\ \end{aligned}$$

If the bracket on the right hand side is multiplied out the lowest order term is quadratic and so leads to the variance. Third order terms are small if *p*(x) is symmetric. The fourth order term corresponds to the variance of the variance. Therefore to second order we have:

139 $$Var\left( {H(x)} \right) = \left( {\left. {\frac{\partial H}{\partial x}} \right|_{{x = \bar{x}}} } \right)^{2} \int {(x - \bar{x})}^{2} p(x) \cdot dx + 4th\;order\;and\;higher\;terms$$

Formally, the next order terms, assuming symmetry around the mean of x, look like:

140 $$\frac{1}{4}\left( {\left. {\frac{{\partial^{2} H}}{{\partial x^{2} }}} \right|_{{x = \bar{x}}} } \right)^{2} \left( {\mu_{4} - \sigma^{4} } \right) + \frac{1}{6}\left( {\left. {\frac{\partial H}{\partial x}} \right|_{{x = \bar{x}}} } \right)\left( {\left. {\frac{{\partial^{3} H}}{{\partial x^{3} }}} \right|_{{x = \bar{x}}} } \right)\mu_{4}$$

Here *µ*_4_ is the fourth moment of the variation from the average. Neglecting these higher order terms we have:

141 $$Var\left( {H(x)} \right) \approx \sigma_{x}^{2} \left( {\left. {\frac{\partial H}{\partial x}} \right|_{{x = \bar{x}}} } \right)^{2}$$

This is the desired result. Note that this result is only true near the mean but that this approximation improves as the sample number, *N*, becomes larger. When *N* is small the actual form of the variance may be quite asymmetric and non-normal.

### Multiple variables

Suppose we have a function *H* of variables *x* and *y*, and we know the standard deviation of both *x* and *y*. As above we have:

142 $$Var\left( {H(x,y)} \right) = \int {\left( {H(x,y) - \overline{{H\left( {x,y} \right)}} } \right)}^{2} p(x,y) \cdot dx$$

If the variables *x* and *y* are independent then *p*(*x*, *y*) = *p*(*x*)*p*(*y*). Futhermore, following the same prescription as above for a single variable and keeping first order terms we arrive at:

143 $$\begin{aligned} &Var\left( {H(x,y)} \right) = \int {\left( {\left( {\left. {\frac{\partial H}{\partial x}} \right|_{{x = \bar{x}}} } \right)\left( {x - \bar{x}} \right) + \left( {\left. {\frac{\partial H}{\partial y}} \right|_{{y = \bar{y}}} } \right)\left( {y - \bar{y}} \right) + higher\;order\;terms} \right)}^{2} p(x)p(y) \cdot dx \hfill \\ &Var\left( {H(x,y)} \right) \approx \sigma_{x}^{2} \left( {\left. {\frac{\partial H}{\partial x}} \right|_{{x = \overline{x} }} } \right)^{2} + \sigma_{y}^{2} \left( {\left. {\frac{\partial H}{\partial y}} \right|_{{y = \bar{y}}} } \right)^{2} \hfill \\ \end{aligned}$$

If the variables are not independent then this first order approximation leads to:

144 $$Var\left( {H\left( {x,y} \right)} \right) \approx \sigma_{x}^{2} \left( {\left. {\frac{\partial H}{\partial x}} \right|_{{x = \bar{x}}} } \right)^{2} + 2cov(x,y)\left( {\left. {\frac{\partial H}{\partial x}} \right|_{{x = \bar{x}}} } \right)\left( {\left. {\frac{\partial H}{\partial y}} \right|_{{y = \bar{y}}} } \right) + \sigma_{y}^{2} \left( {\left. {\frac{\partial H}{\partial y}} \right|_{{y = \bar{y}}} } \right)^{2}$$

The generalization to more variables is straightforward, e.g. for independent variables we have:

145 $$Var\left( {H(x,y,z)} \right) \approx \sigma_{x}^{2} \left( {\left. {\frac{\partial H}{\partial x}} \right|_{{x = \bar{x}}} } \right)^{2} + \sigma_{y}^{2} \left( {\left. {\frac{\partial H}{\partial y}} \right|_{{y = \bar{y}}} } \right)^{2} + \sigma_{z}^{2} \left( {\left. {\frac{\partial H}{\partial z}} \right|_{{z = \bar{z}}} } \right)^{2} \cdots$$

And for dependent variables:

146 $$\begin{aligned} Var\left( {H(x,y,z)} \right) & \approx \sigma_{x}^{2} \left( {\left. {\frac{\partial H}{\partial x}} \right|_{{x = \bar{x}}} } \right)^{2} + \sigma_{y}^{2} \left( {\left. {\frac{\partial H}{\partial y}} \right|_{{y = \bar{y}}} } \right)^{2} + \sigma_{z}^{2} \left( {\left. {\frac{\partial H}{\partial z}} \right|_{{z = \bar{z}}} } \right)^{2} + 2cov(x,y)\left( {\left. {\frac{\partial H}{\partial x}} \right|_{{x = \bar{x}}} } \right)\left( {\left. {\frac{\partial H}{\partial y}} \right|_{{y = \bar{y}}} } \right) \\ \quad + 2cov(x,z)\left( {\left. {\frac{\partial H}{\partial x}} \right|_{{x = \bar{x}}} } \right)\left( {\left. {\frac{\partial H}{\partial z}} \right|_{{z = \bar{z}}} } \right) + 2cov(y,z)\left( {\left. {\frac{\partial H}{\partial y}} \right|_{{y = \bar{y}}} } \right)\left( {\left. {\frac{\partial H}{\partial z}} \right|_{{z = \bar{z}}} } \right) \\ \end{aligned}$$

Note that these expressions are approximations and depend on the nature of the probability distribution function for their applicability. However, they are in common use. The simplest case is when H is a sum of independent variables, in which case we retrieve the usual formula for the propagation of error, i.e.

147 $$Var\left( {H(x,y,z)} \right) \approx \sigma_{x}^{2} + \sigma_{y}^{2} + \sigma_{z}^{2} \cdots$$

In the case of dependent variables we have:

148 $$Var\left( {H(x,y,z)} \right) \approx \sigma_{x}^{2} + \sigma_{y}^{2} + \sigma_{z}^{2} + 2cov\left( {x,y} \right) + 2cov\left( {x,z} \right) + 2cov(y,z)$$

## Appendix 4: Enrichment

Denote:

*A* = total number of actives

*I* = total number of inactives

*a* = number of actives observed

*i* = number of inactives observed

*f* = fraction of inactives observed = *i/I*

*g* = fraction of actives observed = *a/A*

*e* = ROC enrichment

*E* = traditional enrichment

*F* = fraction of the database tested

*s* = *dg*/*df* = slope of the ROC curve at point (*f*, *g*)

By the definition of the ROC Enrichment,

149 $$e = \frac{g}{f}$$

The definition of the traditional enrichment, E is:

150 $$E = \frac{g}{F}$$

Using Eq. 127:

151 $$E = \frac{ef}{F}$$

We also have:

152 $$F = \frac{a + i}{A + I};$$

Using Equation the definition of *i*, the fraction of inactives observed:

153 $$\begin{aligned} F & = \frac{a + If}{A + I} \\ F & = \frac{{\left( \frac{a}{A} \right) + Rf}}{1 + R} = \frac{ef + Rf}{1 + R} \\ F & = f\left( {\frac{e + R}{1 + R}} \right)  \\ \end{aligned}$$

Where *R* is, again, the ratio of inactives to actives. Using Eq. 151 and rearranging leads to:

154 $$E = e\left( {\frac{1 + R}{e + R}} \right)$$

This formula is useful because it gives *E* as a function of *e*. From “Appendix 3”:

$$var(E) = var(e)\left( {\frac{\partial E}{\partial e}} \right)^{2}$$

Using Eq. 154, we have:

155 $$\left( {\frac{\partial E}{\partial e}} \right) = \frac{R(1 + R)}{{\left( {e + R} \right)^{2} }}$$

We would like this formula in the in terms of *E*, rather than *e*. If we rearrange Eq. 154 we see that:

156 $$e = \frac{RE}{1 + R - E}$$

Substituting 156 into 155 we obtain:

157 $$\left( {\frac{\partial E}{\partial e}} \right) = \frac{{\left( {1 + R - E} \right)^{2} }}{R(1 + R)}$$

Thus we arrive at:

158 $$var(E) = var(e)\frac{{\left( {1 + R - E} \right)^{4} }}{{\left( {1 + R} \right)^{2} R^{2} }}$$

Equation 158 still uses the variance of the ROC Enrichment, which depends on e, the ROC Enrichment, and f, the fraction of inactives, which differs from F the fraction in the traditional enrichment:

159 $$var(e) = \frac{1}{f}\left( {\frac{{g\left( {1 - g} \right)}}{A} + \left( {\frac{g}{f}\left( {1 + \frac{\log (e)}{\log (f)}} \right)} \right)^{2} \frac{{f\left( {1 - f} \right)}}{I}} \right)$$

However, both *e* and *f* can be calculated from the traditional enrichment terms, *E* and *F*, namely Eq. 156 for *e* in terms of *E* and *R*. Equation 151 can be rearranged to give:

160 $$f = \frac{EF}{e}$$

Substituting from 156 for *e*, we obtain:

161 $$f = F\frac{(1 + R - E)}{R}$$

Thus, Eqs. 159 and 161 provide all that is necessary to convert Eq. 158 into an equation containing only quantities provided by traditional enrichment, i.e. E, the enrichment, F, the fraction of the total database, A and I, the number of actives and inactives, and the fraction of actives found, (common to both), g.

## Appendix 5: Whether or not to add a noisy system

From Eq. 98 we have:

162 $$Err_{observed,\;N\;systems} = \sqrt {Err_{intrinsic }^{2} + \mathop \sum \limits_{i = 1}^{N} Err_{i}^{2} /\left( {N(N - 1)} \right)}$$

Where,

163 $$Err_{intrinsic }^{2} = \frac{{\sigma_{intrinsic}^{2} }}{N}$$

Suppose we add another system. The question, then, is whether the following statement is true:

164 $$Err_{observed,\,N + 1 systems} < Err_{observed,\,N systems}$$

This is equivalent to:

165 $$\frac{{\sigma_{intrinsic}^{2} }}{N + 1} + \mathop \sum \limits_{i = 1}^{N + 1} Err_{i}^{2} /\left( {N(N + 1)} \right) < \frac{{\sigma_{intrinsic}^{2} }}{N} + \mathop \sum \limits_{i = 1}^{N} Err_{i}^{2} /\left( {N(N - 1)} \right)$$

We have made the assumption here that *σ*_*intrinsic*_ is constant on either side of the equation, i.e. adding a new system does not alter the standard deviation. If *N* is sufficiently large this ought to be a good approximation because the standard deviation is an intrinsic property and because we are using an unbiased estimator for it (i.e. by dividing by *N* − 1, not *N*).

Rearranging gives:

166 $$\frac{{Err_{N + 1}^{2} }}{{N\left( {N + 1} \right)}} + \mathop \sum \limits_{i = 1}^{N} Err_{i}^{2} /\left( {N\left( {N + 1} \right)} \right) - \mathop \sum \limits_{i = 1}^{N} Err_{i}^{2} /\left( {N\left( {N - 1} \right)} \right) < \frac{{\sigma_{intrinsic}^{2} }}{N} - \frac{{\sigma_{intrinsic}^{2} }}{N + 1}$$

167 $$\frac{{Err_{N + 1}^{2} }}{N(N + 1)} - 2\mathop \sum \limits_{i = 1}^{N} Err_{i}^{2} /\left( {(N - 1)N(N + 1)} \right) < \frac{{\sigma_{intrinsic}^{2} }}{N(N + 1)}$$

168 $$\frac{{Err_{N + 1}^{2} }}{N(N + 1)} - 2\frac{{\sigma_{system}^{2} }}{N(N + 1)} < \frac{{\sigma_{{\text{int} rinsic}}^{2} }}{N(N + 1)}$$

Here, *σ*_*system*_ is the standard deviation of the error across all systems. Given that the average of the error ought to be close to zero we have:

169 $$\sigma_{system}^{2} = \frac{1}{N - 1}\mathop \sum \limits_{i = 1}^{N} \left( {Err_{i} - \overline{Err} } \right)^{2} \approx \frac{1}{N - 1}\mathop \sum \limits_{i = 1}^{N} Err_{i}^{2}$$

So the condition that adding the new system reduces the total error in the estimation of the property of interest is:

170 $$\sigma_{intrinsic}^{2} + 2\sigma_{system}^{2} > Err_{N + 1}^{2}$$

## Appendix 6: Key equations

As there are many equations in this article, this appendix provides a summary of the important formulae. Some are standard in the statistical field, some have been reworked here to apply to our field in ways the author has not seen in print, and some are new, such as the work on the error bars of virtual screening enrichment. As such the list below will also detail the provenance of each equation.

Equation 6: *95* *% confidence limits*. Basic definition of a confidence range for a symmetric error, where the *t* statistic is its ‘canonical’ value of 1.96, representing a 95 % confidence interval for large sample size.

Equation 12: *The error of the error.* General formula for the uncertainty in a variance, i.e. a standard deviation squared. This can be used to estimate the error in quantities such as an RMSE, a coefficient of variation, or Cohen’s effect size; any property that depends explicitly on the sample variation.

Equations 18 and 20: *The error in a probability or fractional count*. Standard formula for the standard deviation of a probability (18), and for an integer count, i.e. a fraction of a total count (20).

Equation 21: “*Scale*”-*free form of the expected range of an integer count*.

Equations 22, 23a, 23b: *DeLong formula for AUC variance.* Intended for use when you have primary data, i.e. the complete list of actives and inactives. As per the original paper [19]

Equation 24: *Parametric form of an AUC curve*. Implicit in the work of Hanley, but which the author has made explicit.

Equations 25a, 25b and 25c: *Hanley formula for the variance of an AUC.* Only requiring the AUC, not the primary data. As per the original paper [21]

Equation 26: *Welch*–*Satterthwaite formula for degrees of freedom*. How to calculate the degrees of freedom when there are several sources of variance. As from their papers [25, 26]

Equation 27: *Welch*–*Satterthwaite applied to AUC*. The author’s derivation of a compact form of the Welch–Satterthwaite, using only the AUC and the number of active and inactives.

Equation 28 *: Maximum enrichment with saturation*. A simple formula to bear in mind when using the traditional form of enrichment, i.e. as a fraction of the total database. Author’s form.

Equations 34b and 35b: *Confidence intervals of ROC and traditional enrichment*. To the author’s knowledge these are novel equations for the expected error bounds of both types of enrichment.

Equations 36 and 37: *Definition of covariance and for the best*-*fit slope in linear regression*. Textbook form.

Equations 38, 49: *Variance of best*-*fit slope and intercept.* Textbook form.

Equation 42: *Confidence interval of best*-*fit straight*-*line predictions*. Form that gives the classic “hyperbolic” error bars to prediction, which are small near the center of the data, then grow outside the range of the data. Textbook form.

Equation 45: *Pearson’s r coefficient*. Textbook form.

Equations 49 and 50: *Forward and reverse F transform for Pearson’s r*. Key to calculating confidence ranges for *r*. Transform *r* to F(r), add and subtract standard variance. Transform back to *r* values. Textbook form.

Equation 55: *Population value of r*. Formula to adjust likely bias in the sample version of Pearson’s *r*. Textbook form.

Equations 58, 59a and 59b. *Logit transforms for probability ranges*. Similar to Pearson’s r, except that the variance is calculated from that of the probability (Eq. 18) and the derivative of the logit function (Eq. 59a). Textbook form.

Equation 60: *Analytic form for probability error bars*. Derived by the author, possibly known before but not obviously so.

Equation 74: *Chi* *squared range for standard deviation*. Improved form of estimating asymmetric ranges of a sum of squares that avoids ranges that are less than zero. Requires look-up tables. Textbook form.

Equations 75 and 76: *Variance of compound variables*. Textbook form of how to calculate the variance of a function of one or more variables.

Equations 80 and 81: *Application of Equation* 75 *to a weighted sum*.

Equation 89: *Variance of a ratio of variables*.

Equations 94 and 95: *Application of 89 to Cohen’s d and the Coefficient of Variation*.

Equation 96: *General formula for the addition of known, independent, errors.* Textbook form.

Equation 98: *Total observed error in terms of intrinsic error and ‘system’ error.* A formula that can be used to sharpen the observed error bounds by subtracting out known, “system” errors; for instance, the expected error in the AUC of each system in a test suite. The author’s form.

Equation 101. *Assessment of adding the N* + *1 system*. Determination as to whether adding a new system will increase or decrease the *total* error of a test suite. The author’s form.

Equation 111 and 112. *Variance*-*weighted mean and variance*. How to combine data of the same quantity (*not* an average of different quantities), where the data possesses different variances, for both the mean and the combined variance. Textbook form.

Equation 114. *Variance of a weighted average (of the same quantity).* Textbook form.

Equation 153. *Transforming the ROC Enrichment fraction to the traditional enrichment fraction*. Author’s form.

Equation 154: *Transforming the ROC enrichment into traditional enrichment*. Author’s form.

Equation 156: *Transforming the traditional enrichment into ROC enrichment.* Author’s form.

Equation 161: *Transform the traditional fraction into the ROC fraction.* Author’s form.
